# Supplementary material for: Phase Ib dose-escalation study of the hypoxia-modifier Myo-inositol trispyrophosphate in patients with hepatopancreatobiliary tumors
Source: Nat Commun. 2021 Jun 21;12:3807. doi: 10.1038/s41467-021-24069-w (PMC8217170; doi:10.1038/s41467-021-24069-w)
Supplement: Supplementary file 1 — Supplementary information [file 41467_2021_24069_MOESM1_ESM.pdf]

Phase Ib dose-escalation study of the hypoxia-modifier Myo-inositol trispyrophosphate in patients with hepatopancreatobiliary tumors

Article – clinical phase 1b study

Supplementary Tables & Figures with Legends

Supplementary Table 1 Extended patient baseline characteristics

Supplementary Table 2 Pharmacokinetics of intravenous ITPP administration in individual patients

Supplementary Table 3 Extended patient response and follow-up data

Supplementary Figure 1 Radiological responses stratified by cohort and tumor type.

Supplementary Figure 2 Changes in circulating tumor-specific markers post ITPP and correlation with chemotherapy responses.

Supplementary Figure 3 Changes in circulating angiogenic markers by cohort, tumor type, and radiological responses post-ITPP monotherapy.

Supplementary Figure 4 Correlations within serum markers, tumor markers, and survival.

Supplementary Table 1 Extended patient baseline characteristics

Cohort (Single dose)	Patient number	Age	Gender	Tumor	Tumor localization at study start	ECOG PS	Previous anti-tumor therapies
1 (1866 mg/m2)	1	69	F	PDAC	PAN, LIV, PER	1	Gemcitabine/chloroquine (2x, study combination for improved tolerability of gemcitabine through chloroquine)
	2	70	F	PDAC	PAN, LIV	0	None
	3	80	M	PDAC	RET, LYM, OSS	0	Surgery (total pancreatectomy), paclitaxel/gemcitabine (6x)
	4	70	M	CCC	LIV, PUL, LYM, OSS	0	Cisplatin/gemcitabine (8x), arrow pump implantation & intraarterial floxuridine plus systemic cisplatin/gemcitabine (6x), radiofrequency ablation
2 (3732 mg/m2)	5	60	F	CRLM	LIV, PUL	0	Surgery (rectosigmoid resection), FOLFOXIRI/bevacizumab (4x), arrow pump implantation & intraarterial floxuridine with systemic FOLFIRI (5x)
	6	67	F	PDAC	LIV, PAN, PER, PUL	1	Radiochemotherapy (50Gy with gemcitabine 400mg/m2), gemcitabine/capecitabine (6x)
	7	69	M	CCC	PUL, LIV, PER	0	None
3 (5600 mg/m2)	8	58	M	CRLM	LIV, LYM	1	Surgery (rectal resection, microwave & nano-knife ablation of liver metastases), FOLFOX/cetuximab (6x), FOLFIRI/bevacizumab (4x), FOLFIRI/aflibercept (4x)
	9	51	M	PDAC	LIV, PAN	1	Surgery (Whipple pancreaticoduodenectomy), radiotherapy pancreatic head (5x7Gy), FOLFIRINOX (10x)
	10	69	M	CCC	LIV	1	Gemcitabine/cisplatin (6x), arrow pump implantation & intraarterial floxuridine
4 (7000 mg/m2)	11	71	M	CCC	PER	1	Surgery (extended right hemi-hepatectomy)
	12	50	M	HCC (HepB)	LIV	1	None
	13	65	M	HCC (HepC)	LIV	1	Surgery (liver wedge resection), multiple selective internal radiation therapy (Yttrium 90)
	14	38	M	HCC (C2)	LIV	1	Surgery (right hemi-hepatectomy), sorafenib (5 months)
5 (8750 mg/m2)	15	43	F	PDAC	PUL, LIV, PER	0	Surgery (Whipple pancreaticoduodenectomy, wedge resections lung metastases), gemcitabine (6x), cyberknife therapy (liver metastases)
	16	77	M	CCC	LIV, LYM	0	Gemcitabine/cisplatin (4x), gemcitabine/oxaliplatin (6x), capecitabine/fluorouracil/leucovorin (4x)
	17	65	M	PDAC	PAN, LIV	1	FOLFIRINOX (10x), Nab-paclitaxel/gemcitabine (3x)
6 (10500 mg/m2)	18	41	F	CRLM	PUL, LIV, LYM	0	Surgery (sigmoid resection, two-stage extended right hemi-hepatectomy), FOLFOX/cetuximab (17x)
	19	66	M	CCC	LIV, LYM	0	Surgery (right hemi-hepatectomy right, liver wedge resection), gemcitabine/cisplatin (3x)
	20	75	M	CRLM	LIV	0	Surgery (rectosigmoid resection, liver wedge resections & microwave ablation), FOLFOX (12x), capecitabine (3x), FOFIRI/cetuximab (5x)
	21	61	F	CRLM	LIV, PUL	0	Surgery (right hemicolectomy, left hemi-hepatectomy, microwave ablation), FOLFIRI/bevacizumab (6x), nano-knife ablation liver metastases, capecitabine/bevacizumab (14x), radiotherapy 5x7Gy liver hilus, FOLFOXIRI/bevacizumab (7x), selective internal radiation therapy (Yttrium 90) of liver metastases.
7 (12390 mg/m2)	22	54	F	CRLM	LIV, PER	1	Oxaliplatin/capecitabine/bevacizumab (4x), FOLFOX/bevacizumab (4x), radioembolization, panitumumab, TAS102/Lonsurf, FOLFIRI (4x)
	23	48	F	CRLM	LIV, PUL	0	FOLFOXIRI/bevacizumab (16x), nano-knife ablation liver metastases
	24	66	M	PDAC	PAN, LIV	0	Surgery (liver wedge resection), FOLFIRINOX (2x)
8 (14500 mg/m2)	25	68	M	PDAC	PAN, LIV	0	Surgery (Whipple pancreaticoduodenectomy), gemcitabine (6x), cisplatin/gemcitabine (6x)
	26	49	M	PDAC	PAN, LIV	1	FOLFIRINOX (15x), paclitaxel/gemcitabine (6x)
	27	55	F	CCC	LIV, PUL	0	Panhepatic transarterial radioembolization
	28	58	M	CRLM	LIV, PER	0	Surgery (rectosigmoid resection), FOLFOX/bevacizumab (12x), FOLFIRI/bevacizumab (5x)

Abbreviations:
Gender: F = Female, M = Male; Tumor: PDAC = Pancreatic ductal adenocarcinoma, CCC = Cholangiocarcinoma, CRLM = colorectal liver metastases, HCC = hepatocellular carcinoma
ECOG PS = Eastern Cooperative Oncology Group performance status
Localization: PAN = Pancreas, LIV = Liver, PER = Peritoneum, RET = Retroperitoneum, LYM = Lymph nodes, OSS = Bones, PUL = Lung
Previous therapies: x indicates the number of cycles received of the respective chemotherapy regimen, Gy = Gray (J/kg)
HepB = chronic hepatitis B; HepC = chronic hepatitis C; C2 = Alcohol abuse
FOLFOX = leucovorin, fluorouracil, oxaliplatin; FOLFIRI = leucovorin, fluorouracil, irinotecan; FOLFIRINOX = leucovorin, fluorouracil, irinotecan, oxaliplatin

Supplementary Table 2 Pharmacokinetics of intravenous ITPP administration of individual patients.

Cohort (Single dose)	Patient Number	Height [cm]	Weight [kg]	BMI [kg/m2]	BSA [m2] (DuBois/DuBois)	Absolute individual dose per infusion	Average infusion speed [ml/h]	C_{min} [mg/L]	C_{max} [mg/L]	C_{max} / Dose [mg/L/mg]	T_{max} [h]	C_{last} [mg/L]	T_{last} [h]	Half life / $T_{0.5}$ [h]	Elimination rate λz [/h]	AUC_{Tmax} [h*mg/L]	AUC_{∞} [h*mg/L]	AUC_{∞} / Dose [h*mg/L/mg]	$AUMC_{Tmax}$ [h2*mg/L]	$AUMC_{\infty}$ [h2*mg/L]	MRT_{Tmax} [h]	MRT_{∞} [h]	VZO [L]	CLO [L/h]	VSSO [L]
1 (1866 mg/m2)	1	148	45	21	1.36	2594	125	0	54.7	0.0	6.0	33.9	9	N.A.	N.A.	349.1	N.A.	N.A.	1904.2	N.A.	1.5	N.A.	N.A.	N.A.	N.A.
	2	150	72	32	1.67	3284	125	0	45.8	0.0	6.0	15.7	10	1.3	0.5	338.2	366.8	0.2	1863.8	2201.9	1.5	2.0	9.3	5.1	10.2
	3	176	63	20	1.77	3312	125	0	55.3	0.0	6.0	46.6	10	N.A.	N.A.	372.1	N.A.	N.A.	2050.4	N.A.	1.5	N.A.	N.A.	N.A.	N.A.
	4	170	73.5	25	1.85	3471	125	0	42.2	0.0	8.5	22.8	10	N.A.	N.A.	311.3	N.A.	N.A.	1825.7	N.A.	1.9	N.A.	N.A.	N.A.	N.A.
2 (3732 mg/m2)	5	160	74	29	1.77	6717	129	0	106.3	0.0	6.0	40.6	10	1.4	0.5	745.9	826.4	0.2	4278.4	5242.1	1.7	2.3	8.9	4.5	10.6
	6	157	57.4	23	1.57	5897	131	0	106.7	0.0	6.0	52.1	10	1.8	0.4	761.5	896.6	0.2	4442.2	6142.5	1.8	2.9	10.8	4.2	11.9
	7	184	99	29	2.22	8397	132	0	148.7	0.0	6.0	69.7	10	1.8	0.4	1032.3	1215.3	0.3	6030.7	8341.9	1.8	2.9	8.1	3.1	8.8
3 (5600 mg/m2)	8	179	83	26	2.02	11256	130	0	163.0	0.0	6.0	50.5	10	1.7	0.4	1142.0	1264.3	0.2	6061.8	7579.9	1.3	2.0	10.7	4.4	8.8
	9	184	63	19	1.83	10024	132	0	120.7	0.0	6.0	45.2	10	1.4	0.5	856.4	945.9	0.2	4859.5	5932.4	1.7	2.3	11.7	5.9	13.4
	10	165	96	35	2.03	11340	133	0	246.0	0.0	6.0	94.2	10	1.5	0.5	1779.2	1987.5	0.4	9884.9	12427.3	1.6	2.3	6.2	2.8	6.3
4 (7000 mg/m2)	11	173	78	26	1.92	13580	133	0	283.0	0.0	9.0	214.0	10	5.7	0.1	1942.5	3708.1	0.5	11888.0	44109.2	2.1	7.9	15.6	1.9	14.9
	12	180	62	19	1.79	12222	133	0	166.5	0.0	6.0	94.0	10	2.2	0.3	1258.2	1559.8	0.2	7283.0	11265.8	1.8	3.2	14.4	4.5	14.5
	13	174	103	34	2.17	15617	133	0	224.5	0.0	3.0	125.5	10	2.6	0.3	1667.1	2130.6	0.3	9303.5	15649.8	1.6	3.3	12.1	3.3	11.0
	14	184	81	24	2.04	14245	134	0	292.0	0.0	6.0	43.0	10	1.3	0.5	1676.0	1755.9	0.3	8707.6	9654.5	1.2	1.5	7.4	4.0	6.0
5 (8750 mg/m2)	15	160	45	18	1.44	12372	135	0	266.5	0.0	6.0	81.5	10	1.5	0.4	1729.5	1910.9	0.2	9560.8	11777.2	1.5	2.2	10.2	4.6	9.9
	16	173	84	28	1.98	17578	134	0	306.0	0.0	6.0	168.0	10	2.3	0.3	2397.4	2946.9	0.3	13507.9	20800.6	1.6	3.1	9.7	3.0	9.1
	17	178	68	21	1.85	16047	134	0	201.0	0.0	6.0	101.0	10	2.6	0.3	1424.5	1803.3	0.2	8120.6	13329.9	1.7	3.4	18.2	4.9	16.5
6 (10500 mg/m2)	18	159	55	22	1.56	16369	134	0	265.0	0.0	6.0	74.8	10	1.0	0.7	1813.1	1921.1	0.2	10264.8	11500.0	1.7	2.0	7.9	5.5	10.9
	19	174	78	26	1.93	20391	137	0	339.5	0.0	3.0	185.0	10	4.0	0.2	2491.5	3551.0	0.3	13653.6	30316.3	1.5	4.5	16.9	3.0	13.4
	20	178	102	32	2.20	23583	138	0	451.0	0.0	6.0	337.5	10	11.0	0.1	3266.4	8601.3	0.8	19354.9	157035.6	1.9	14.3	19.3	1.2	17.4
	21	163	50	19	1.52	15970	145	0	299.5	0.0	3.0	148.5	10	2.0	0.3	2233.0	2661.5	0.3	12411.4	17933.0	1.6	2.7	11.4	3.9	10.8
7 (12390 mg/m2)	22	163	77	29	1.83	22648	144	0	367.5	0.0	6.0	177.5	10	2.0	0.4	2548.8	3050.7	0.2	14937.6	21377.1	1.9	3.0	11.5	4.1	12.2
	23	169	57	20	1.65	20455	152	0	403.5	0.0	6.0	152.0	10	1.3	0.5	2725.4	3012.0	0.2	15888.2	19295.7	1.8	2.4	7.8	4.1	9.9
	24	193	83	22	2.13	26427	147	0	311.0	0.0	6.0	129.0	10	1.8	0.4	2231.0	2566.2	0.2	12461.8	16684.7	1.6	2.5	12.5	4.8	12.1
8 (14500 mg/m2)	25	176	75	24	1.91	27709	140	0	496.0	0.0	6.0	220.5	10	1.6	0.4	3458.0	3978.5	0.3	20144.2	26577.3	1.8	2.7	8.6	3.6	9.8
	26	183	78	23	2.00	28985	143	0	377.0	0.0	6.0	111.5	10	1.3	0.5	2612.8	2818.3	0.2	14245.4	16679.9	1.5	1.9	9.5	5.1	9.9
	27	173	98	33	2.11	30667	142	0	464.5	0.0	6.0	226.5	10	2.3	0.3	3213.9	3977.9	0.3	18540.8	28758.2	1.8	3.2	12.3	3.6	11.8
	28	179	120	37	2.36	34249	143	0	426.0	0.0	6.0	125.7	10	1.7	0.4	2461.1	2763.6	0.2	14081.6	17835.7	1.7	2.5	12.6	5.2	12.9

Abbreviations:
BSA = Body surface area; BMI = Body mass Index; C_{min} [mg/L] = minimal concentration at start of infusion; C_{max} [mg/L] = maximum concentration; C_{max} / Dose [mg/L/mg] = dose normalized Cmax;
T_{max} [h] = time of maximum concentration; C_{last} [mg/L] = last positive concentration observed; T_{last} [h] = time of last positive concentration; Half-life / $T_{0.5}$ [h] = half-life by lambda z = ln(2)/LAMZ;
Elimination rate λ_z [/h] = lambda z negative of best fit terminal slope; AUC_{Tmax} [h*mg/L] = AUC from 0 to T_{max} ; AUC_{∞} [h*mg/L] = AUC infinity observed; AUC_{∞} / Dose [h*mg/L/mg] = AUC infinity / Dose;
$AUMC_{Tmax}$ [h2*mg/L] = AUMC to the T_{last} ; $AUMC_{\infty}$ [h2*mg/L] = AUMC infinity observed using C_{last} ; MRT_{Tmax} [h] = mean residence time (MRT) to T_{last} for intravascular administration;
MRT_{∞} [h] = mean residence time (MRT) infinity using C_{last} for intravascular administration; VZO [L] = volume of distribution determined by elimination rate λ_z and AUC_{∞} for intravascular administration; CLO [L/h] = clearance using AUC_{∞} for intravascular administration; VSSO [L] = volume of distribution at steady state using C_{last} for intravascular administration

Supplementary Table 3 Extended patient response & follow-up data

Cohort (Single dose)	Patient number	Tumor	Nr. of ITPP Infusion received	Primary study endpoint reached?	Radiological response after ITPP		Follow up therapy	Radiological response after chemotherapy		Further therapy	Follow up* from study start [days]	Survival status*
					RECIST 1.1	EORTC		RECIST 1.1	EORTC			
1 (1866 mg/m2)	1	PDAC	2	NO – Dropout	N.A.	N.A.	None	N.A.	N.A.	None	16	Dead
	2	PDAC	9	YES – Regular	PD	PMD	FOLFIRINOX (6x)	SD	PMR	FOLFIRINOX (7x), FOLF (8x), nab-paclitaxel/gemcitabine (3x)	464	Dead
	3	PDAC	9	YES – Regular	SD	PMR	Gemcitabine (6x)	SD	N.A.	none	159	Dead
	4	CCC	9	YES – Regular	SD	SMD	FOLFIRINOX (10x)	SD	PMR	Palliative radiotherapy (30Gy), systemic cisplatin/cemcitabine (1x) and intraarterial floxuridine	398	Dead
2 (3732 mg/m2)	5	CRLM	9	YES – Regular	SD	SMD	FOLFIRI/ bevacizumab (5x)	SD	SMD	Fluorouracil/bevacizumab (4x), surgery (wedge resection lung metastases), FOLFIRI/aflibercept (11x), regorafenib (3x)	783	Dead
	6	PDAC	9	YES – Regular	PD	SMD	FOLFIRINOX (7x)	SD	PMR	None	188	Dead
	7	CCC	9	YES – Regular	SD	PMR	Carboplatin/ gemcitabine (6x)	N.A.	N.A.	Capecitabine/oxaliplatin (2x)	158	Unknown
3 (5600 mg/m2)	8	CRLM	9	YES – Regular	PD	SMD	FOLFOX (4x)	PD	SMD	Trifluridin/iipiracil (2 weeks)	159	Dead
	9	PDAC	9	YES – Regular	PD	PMR	Nab-paclitaxel /gemcitabine (4x)	PD	PMD	Nal-Irinotecan/leucovorin/fluorouracil (1x)	171	Dead
	10	CCC	9	YES – Regular	SD	SMD	Cisplatin/ gemcitabine (2x)	SD	N.A.	FOLFIRI (5x)	206	Dead
4 (7000 mg/m2)	11	CCC	8	YES – Regular	PD	PMD	Gemcitabine/ carboplatin (1x)	N.A.	N.A.	None	193	Dead
	12	HCC	9	YES – Regular	SD	SMD	Sorafenib (2 months)	N.A.	N.A.	None	219	Unknown
	13	HCC	8	YES – Regular	SD	SMD	Sorafenib (3 months)	PR	SMD	Sorafenib (4 months), regorafenib (1 months), nivolumab (2 months), cabozantinib (2x)	684	Dead
	14	HCC	7	YES – Regular	N.A.	N.A.	None	N.A.	N.A.	None	10	Unknown
5 (8750 mg/m2)	15	PDAC	9	YES – Regular	SD	SMD	FOLFIRINOX (6x)	PD	PMD	Palliative radiotherapy (30Gy)	196	Dead
	16	CCC	9	YES – Regular	PD	PMD	Capecitabin/ fluorouracil/ leucovorin (3x)	PD	PMD	Olaparib (2x)	202	Dead
	17	PDAC	6	YES – Regular	PD	PMD	Nal-irinotecan/ fluorouracil (2x)	N.A.	N.A.	None	68	Dead
6 (10500 mg/m2)	18	CRLM	8	YES – Regular	PD	PMD	FOLFOX/ bevacizumab (3x)	PD	PMD	FOLFOX/bevacizumab (2x)	394	Dead
	19	CCC	9	YES – Regular	SD	SMD	Herceptin/ FOLFIRI (6x)	PR	PMR	Herceptin/FOLFIRI (3x), stereotactic radiotherapy of 2 liver metastases (45Gy each)	510	Alive
	20	CRLM	9	YES – Regular	PD	PMD	FOLFIRI/ cetuximab (4x)	SD	N.A.	Cetuximab (8 months), capecitabine/irinotecan (1x), trifluridin/tipiracil (2 months)	500	Alive
	21	CRLM	9	YES – Regular	SD	PMD	Regorafenib (2 months)	SD	SMD	Microwave ablation liver metastases, trifluridin/tipiracil (1 month)	301	Dead

7 (12390 mg/m2)	22	CRLM	7	YES – Regular	N.A.	N.A.	None	N.A.	N.A.	None	87	Dead
	23	CRLM	9	YES – Regular	PD	PMD	FOLFIRI/ aflibercept (10x)	SD	SMD	Regorafenib (4 months)	380	Dead
	24	PDAC	9	YES – Regular	SD	PMR	FOLFIRINOX (1x)	SD	N.A.	Palliative radiotherapy (39Gy)	392	Alive
8 (14500 mg/m2)	25	PDAC	9	YES – Regular	PD	PMD	Leucovorin/ fluorouracil (1x)	N.A.	N.A.	None	43	Dead
	26	PDAC	9	YES – Regular	SD	SMD	Nal-irinotecan/ fluorouracil (3x)	PD	N.A.	None	73	Dead
	27	CCC	8.5	YES – Regular	SD	PMD	Gemcitabine/ cisplatin (3x)	SD	SMD	Gemcitabine/cisplatin (4x)	266	Alive
	28	CRLM	9	YES – Regular	SD	SMD	FOLFIRI/ bevacizumab (4x)	SD	SMD	FOLFIRI/bevacizumab (1x), trifluridin/tipiracil (1 month)	206	Dead

Abbreviations:
Tumor: PDAC = Pancreatic ductal adenocarcinoma, CCC = Cholangiocarcinoma, CRLM = colorectal liver metastases, HCC = hepatocellular carcinoma
N.A. = Not available
RECIST 1.1 criteria: CR = complete response, PR = partial response, SD = stable disease, PD = progressive disease
EORTC criteria: CMR = complete metabolic response, PMR = partial metabolic response, SMD = stable metabolic disease, PMD = progressive metabolic disease

Follow up after ITPP unavailable for patient Nr. 14 due to leaving the country prematurely for inclusion in another phase 1 trial.
Follow up after ITPP unavailable for patient Nr. 22 due to switch to best supportive care and wish to not undergo further exams.
*Follow-up and survival last assessed per 31st of December 2018.

FOLFOX = leucovorin, fluorouracil, oxaliplatin; FOLFIRI = leucovorin, fluorouracil, irinotecan; FOLFIRINOX = leucovorin, fluorouracil, irinotecan, oxaliplatin

Supplementary Figure 1

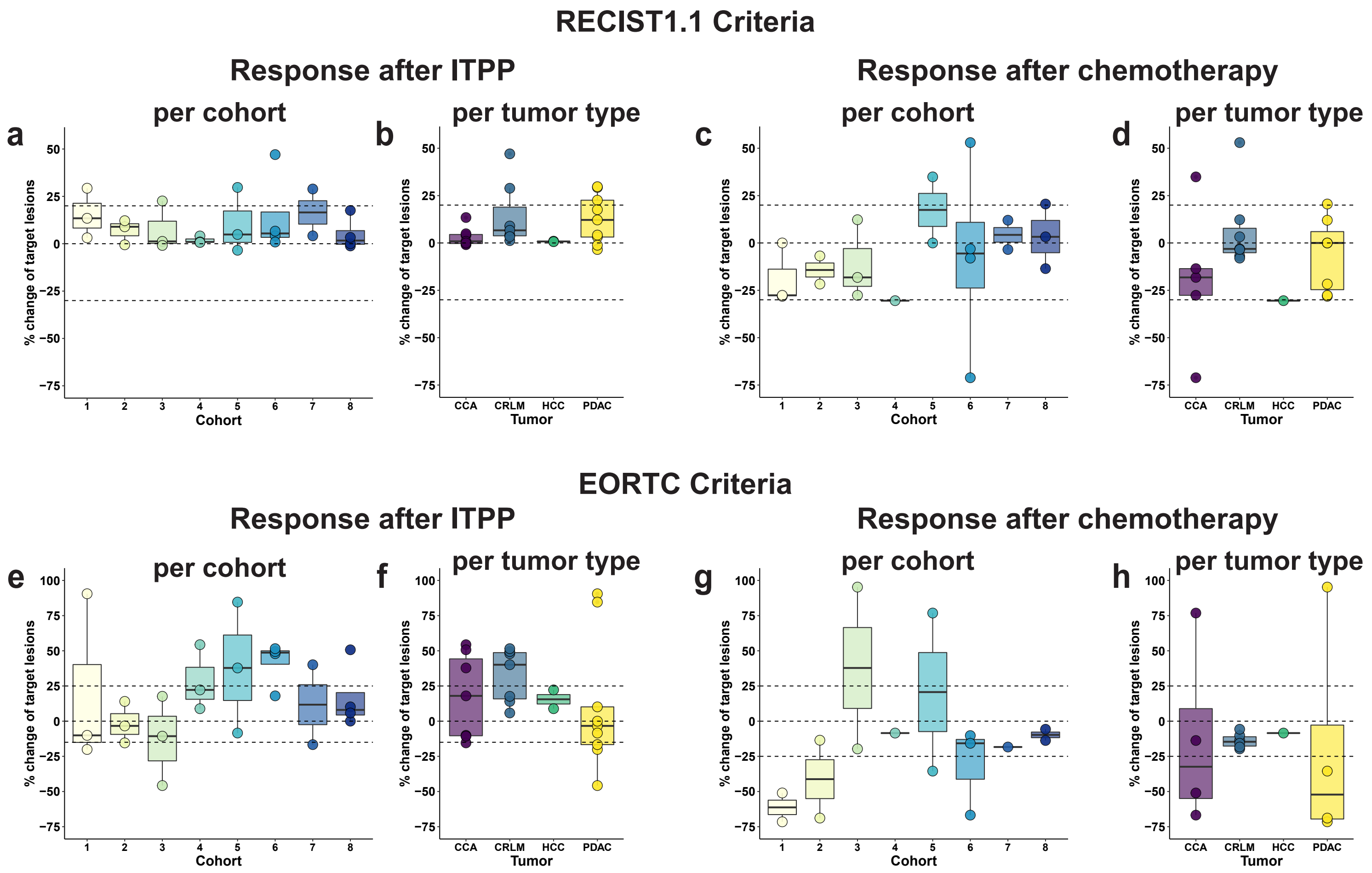

Supplementary Fig. 1 Radiological responses stratified by cohort and tumor type. Response in radiological target lesions according to RECIST1.1 stratified by cohort (**a**) and tumor type (**b**) after ITPP monotherapy and by cohort (**c**) and tumor type (**d**) after chemotherapy. Response in radiological target lesions according to EORTC stratified by cohort (**e**) and tumor type (**f**) after ITPP monotherapy and by cohort (**g**) and tumor type (**h**) after chemotherapy. Boxplots display median values with the upper and lower ends representing the 25th and 75th quartiles, respectively. Whiskers extend to values within 1.5 * IQR from the boxplot, with all individual data points shown overlaid and colored according to either cohort or tumor type. N=27 individual patients.

Supplementary Figure 2

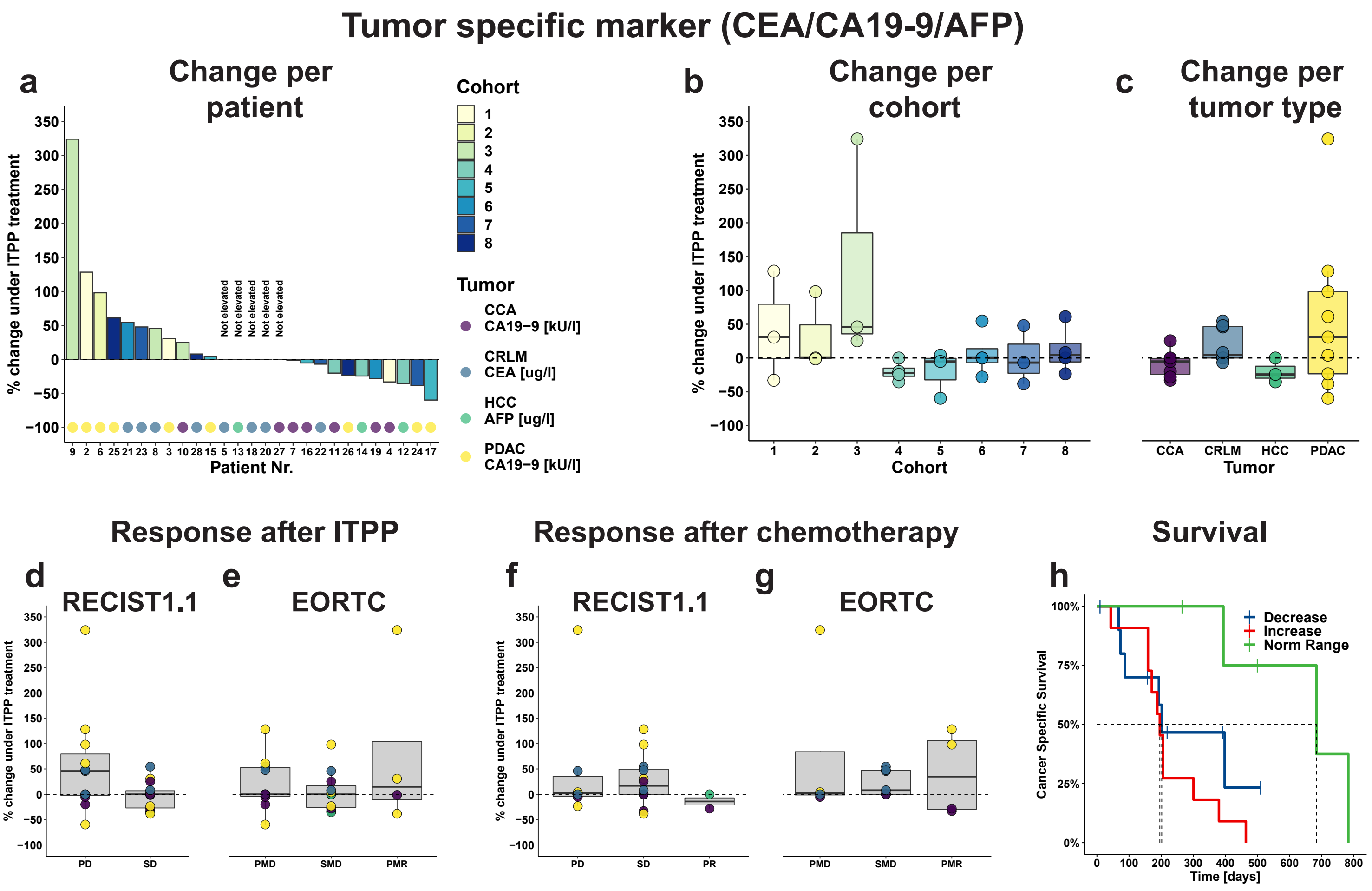

Supplementary Fig. 2 Changes in circulating tumor-specific markers post ITPP and correlation with chemotherapy responses. Changes in tumor-specific markers (CEA for CRLM, AFP for HCC, and CA19-9 for CCC and PDAC) under ITPP treatment are shown as waterfall plot per patient (**a**), or stratified by cohort (**b**), tumor type (**c**), RECIST (**d**), and EORTC (**e**) responses after ITPP monotherapy, and by RECIST (**f**) and EORTC (**g**) responses after chemotherapy as boxplots displaying median values with the upper and lower ends representing the 25th and 75th quartiles, respectively. Whiskers extend to values within 1.5 * IQR from the boxplot, with all individual data points shown overlaid and colored according to cohort or tumor type. Percentage changes relate to pre- versus post-ITPP monotherapy marker levels. (**h**) Survival displayed as Kaplan-Meier curves stratified by decreasing, increasing, or unaltered marker levels within normal range. N=27 individual patients, marker measurements performed as technical duplicates.

Supplementary Figure 3

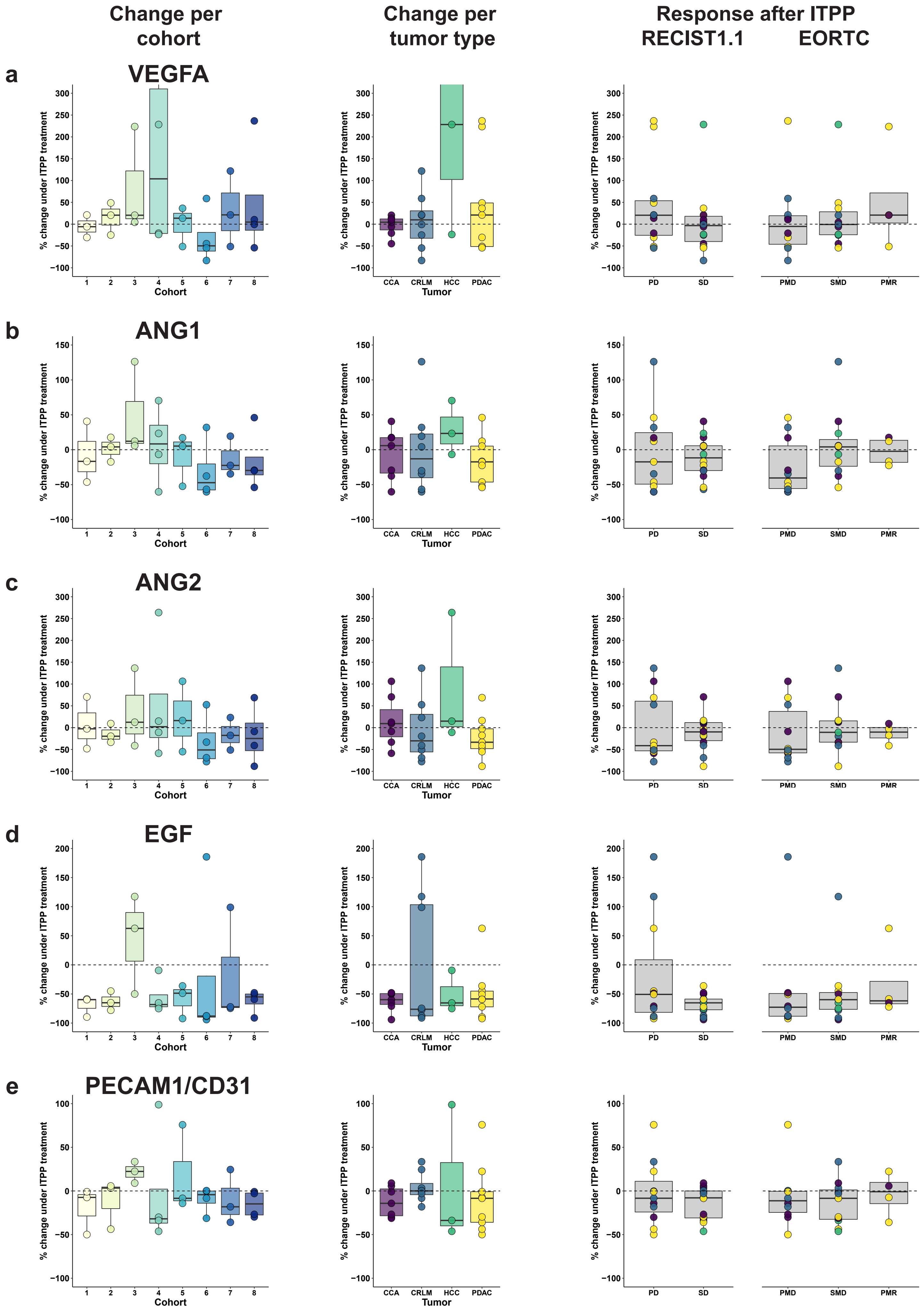

Supplementary Fig. 3 Changes in circulating angiogenic markers by cohort, tumor type, and radiological responses post-ITPP monotherapy. Changes in circulating levels of VEGFA (**a**), ANG1 (**b**), ANG2 (**c**), EGF (**d**), and PECAM1/CD31 (**e**). Percentage changes relate to pre- versus post-ITPP monotherapy marker levels and are shown (from left to right) by cohort, tumor type, and RECIST/EORTC responses after ITPP monotherapy. Boxplots display median values with the upper and lower ends representing the 25th and 75th quartiles, respectively. Whiskers extend to values within 1.5 * IQR from the boxplot, with all individual data points shown overlaid and colored according to either cohort or tumor type. N=27 individual patients, marker measurements performed as technical duplicates.

Supplementary Figure 4

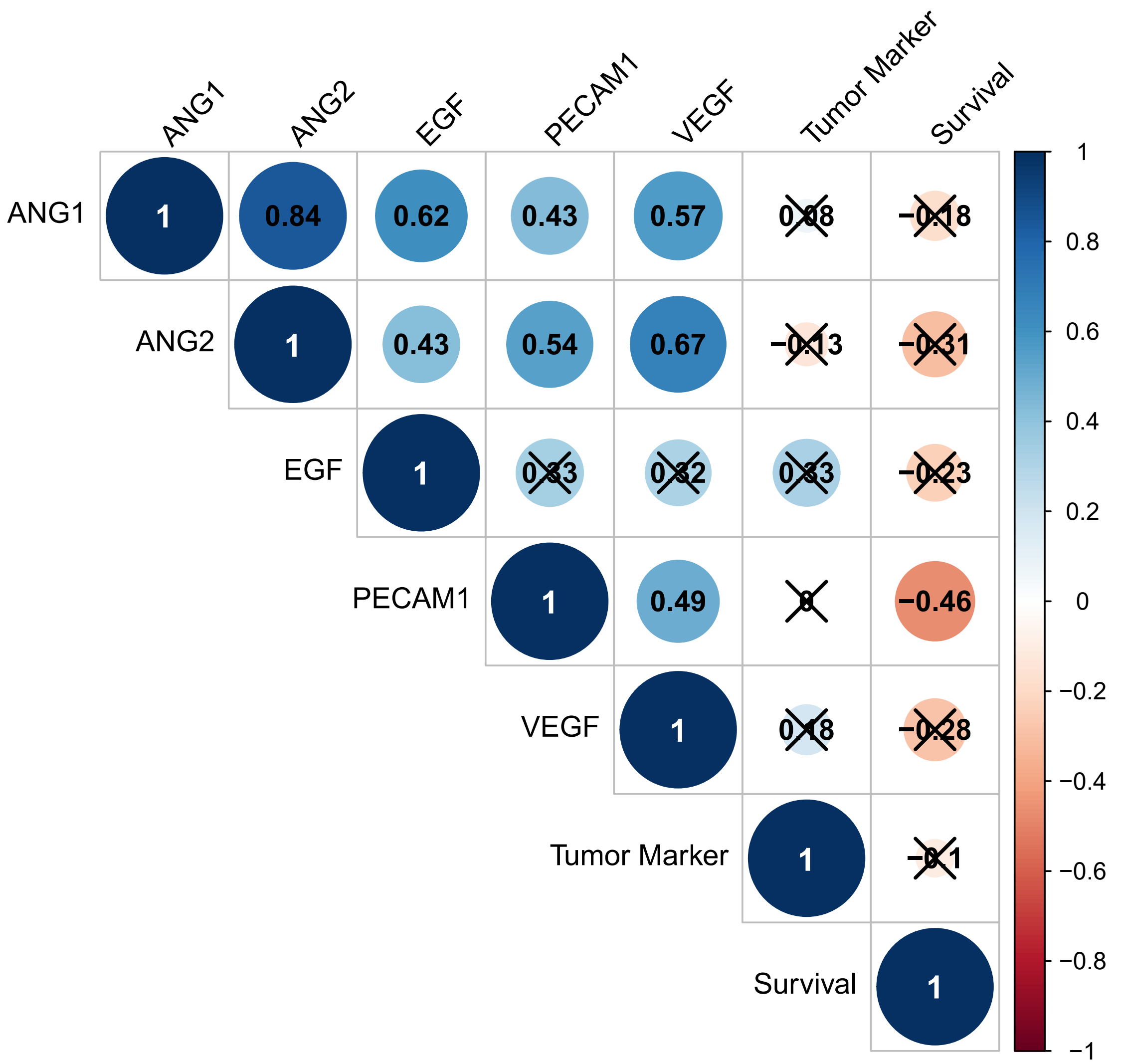

Supplementary Fig. 4 Correlations within serum markers, tumor markers, and survival. Correlations between individual patient changes (n=27) before versus after ITPP monotherapy of angiogenic markers (VEGFA, ANG1, ANG2, EGF, PECAM1), tumor-specific markers (either CEA, CA19-9, or AFP), and survival. Blue circles indicate positive correlations, red circles indicate negative correlations, and circle sizes reflect correlation strength. Numbers within circles refer to Pearson's correlation coefficients. Insignificant ($P > 0.05$) coefficients are crossed out. No adjustment for multiple testing was implemented. Angiogenic molecules showed significant inter-marker correlations except for EGF vs VEGF and PECAM1, likely because of the dominant EGF decrease.

Phase Ib dose-escalation study of the hypoxia-modifier Myo-inositol trispyrophosphate in patients with hepatopancreatobiliary tumors

Article – clinical phase 1b study

Supplementary Note 1: Study Protocol

Table of Contents

Supplementary Note 1: Study Protocol 2
Study registrations 2
List of accepted versions and amendments of the study protocol 2
Background and rationale of the study 3
Investigational product and indication 3
Preclinical evidence to date 3
Clinical evidence to date 4
General study course 5
Dose escalation and dose rationale 6
Risk–benefit considerations 9
Study objectives 10
Study outcomes 11
Study population 12
Inclusion criteria 12
Exclusion criteria 13
Justification for choice of study population 13
Recruitment and screening 13
Criteria for withdrawal/discontinuation of participants 14
Experimental intervention 15
Safety 16
Definition of (S)AEs and other safety-related events 16
Recording of (S)AEs and other safety-related events 17
Assessment of (S)AEs and other safety-related Events 18
Reporting of (S)AEs and other safety-related Events 19
Statistical methods 20
R Session information for statistical analyses 21
Supplementary references 22

Supplementary Note 1: Study Protocol

Note: The "Supplementary Note 1: Study Protocol" is adopted in part from the currently accepted study protocol (Version 6, Amendment 1, April 25, 2016) as approved on June 16, 2016, by the responsible independent ethics committee of the canton of Zurich (KEK-ZH-Nr. 2014-0374) and the Swiss regulatory authorities Swissmedic (2015DR1009). Essential parts of the study protocol have been previously published[1].

Study registrations

Title	A PHASE IB, SINGLE-CENTER STUDY OF THE EFFECTS OF OXY111A IN PRIMARY AND SECONDARY HEPATO-PANCREATO-BILIARY NEOPLASM
Abbreviation	OXY1A clinical trial
Cantonal ethics committee of the canton of Zurich, Switzerland (BASEC number)	KEK-ZH-Nr.2014-0374 (PB_2016-01183)
Swissmedic	2015DR1009
ClinicalTrials.gov	NCT02528526

Note: OXY1A/OXY111A refers to the brand name of ITPP, as marketed by the company NormOxys.

List of accepted versions and amendments of the study protocol

Version of study protocol	Date of version	Date of submission	Date of approval by responsible ethics committee
Version 1.0	21.07.2014	22.07.2014	09.09.2014
Version 2.0	12.09.2014	12.09.2014	16.09.2014
Version 3.0	19.09.2014	19.09.2014	03.10.2014
Version 4.0	06.10.2014	06.10.2014	17.10.2014
Version 5.0	23.12.2014	23.12.2014	07.01.2015
Version 6.0 (Amendment)	01.03.2015	01.03.2015	08.05.2015
Version 6.1 (Amendment)	25.04.2016	02.05.2016	16.06.2016

Background and rationale of the study

Hypoxia occurs in almost any solid tumor beyond a certain size and is key to the development and progression of disease. Decreased oxygen partial pressure modifies tumor behavior, with a resulting increase in cancer-associated angiogenesis being the most well-recognized effect[2, 3]. Novel anti-cancer agents (e.g. bevacizumab) have been designed that specifically target the angiogenic response with the aim to starve the tumor. However, angiogenesis is only one part of the tumor's response to hypoxia.

Molecularly, hypoxia leads to the stabilization of hypoxia-inducible factors (HIFs), transcription factors that induce a transcriptional program underlying the hypoxic response. Apart from angiogenesis (e.g. via vascular endothelial growth factor [VEGF]), HIFs promote several processes that foster tumor development, including inflammation (e.g. via the NF-κB pathway), a shift from oxidative phosphorylation to glycolysis (the Warburg effect, e.g. via upregulation of glucose transporter), invasive behavior (e.g. via Twist, an inducer of the epithelial-to-mesenchymal transition), and malignant potential (e.g. via OCT4 and other genes promoting a stem cell phenotype). Furthermore, HIFs have been implicated in suppressing adaptive immunity and protecting against cell death. Of note, hypoxia predicts a poor outcome with most tumor types[4], consistent with the view that it promotes aggressive cancer behavior. Therefore, prevention of hypoxia may provide an anticancer strategy that is superior to existing anti-angiogenic approaches[5, 6].

Investigational product and indication

Myo-inositol trispyrophosphate (ITPP; brand name: OXY111A) is a synthetic allosteric effector of hemoglobin[7, 8]. Red blood cells take up ITPP via the band 3.1 transporter[9], where the drug promotes the dissociation of oxygen from hemoglobin under low pO_2.[10] In other words, ITPP increases the oxygen-releasing capacity of hemoglobin specifically under hypoxic conditions and thus has the potential to prevent hypoxia without affecting oxygen levels in the surrounding normoxic cells. Of note, ITPP is the first anti-hypoxic compound without known, significant toxicities[11].

Preclinical evidence to date

ITPP has been tested in several animal models of cancer.[10, 12, 13, 14, 15, 16, 17, 18] In a syngeneic, orthotopic rat model of hepatocellular carcinoma, ITPP prevented Hif1α stabilization as well as Vegf production and increased the apoptotic index, leading to a rapid reduction in tumor load compared to untreated animals[10]. All control animals died within 80 days, while nine out of ten ITPP -treated rats were still alive after 100 days without evidence of a detectable tumor mass on micro-computed tomography (CT) imaging. In comparison, only one out of ten rats survived to day 100 following treatment with the conventional cytotoxin doxorubicin. ITPP did not affect hematologic parameters, and no side effects were observed. Furthermore, no side effects have been apparent in any animal models, including those in which ITPP improved exercise capacity in healthy and sick mice[11]. Because of this effect, ITPP is being used as a doping agent in horse races. These outcomes in nonhuman animals further suggest that ITPP might improve performance in humans who are unwell[19].

Although ITPP also prolonged survival in a syngeneic melanoma mouse model[14], an improvement in oxygenation alone is not expected to have curative potential. In a xenogeneic mouse model of human colon carcinomatosis, ITPP treatment did not eliminate malignancy but was still more efficient than capecitabine in decreasing tumor growth[12]. Our results are consistent with these latter findings. In a newly developed syngeneic mouse model of colorectal liver metastasis, ITPP extended survival by 60%. In parallel, we observed an inhibition of HIF

activation, a shift from glycolysis to oxidative phosphorylation, reduced levels of inflammatory markers, lower expression of cancer stem cell markers, and inhibition of the epithelial-to-mesenchymal transition, along with reduced invasiveness following ITPP treatment[16, 17]. The available evidence fully supports an efficient inhibition of the hypoxic tumor response through ITPP.

Of note, a postulated feature that results from increased intratumoral pO_2 is the normalization of tumor-associated vessels. The tumor vasculature is porously structured and leaky, leading to suboptimal tumor perfusion and consequently to persistent or even intermittent hypoxia, as well as insufficient delivery of therapeutic agents. In the two reports in which vasculature was analyzed[12, 14], tumor vessels regained integrity and impermeability following ITPP exposure.

The concept of vessel normalization through ITPP has been confirmed in a syngeneic rat model of pancreatic carcinoma[15] and is directly relevant to patients in our study. In brief, ITPP treatment alone restricted primary growth and metastasis formation. Consequently, survival increased from 52 days in control animals to 93 days following treatment (compared to 63 days in rats treated with 100 mg/kg gemcitabine, the standard treatment for pancreatic cancer at the time of the study). Based on the observed ITPP-induced normalization of the tumor-associated vasculature, the investigators reasoned that ITPP should also enhance the delivery and thus the efficacy of gemcitabine. An initial attempt to use ITPP combined with gemcitabine failed because of toxicity-related mortality, suggesting that gemcitabine was highly active and would require down-dosing. We were, however, intrigued to note that following exposure to ITPP combined with half the usual dose of gemcitabine, all animals survived beyond 200 days. Autopsy revealed an absence of tumors in these rats, confirming the cure of experimental pancreatic cancer through the combined treatment. Thus, ITPP greatly enhanced the efficacy of a drug that has little impact on its own.

Using our syngeneic mouse model of colorectal liver metastasis, we confirmed the potentiation of chemotherapy efficacy through ITPP[16]. Survival was extended with use of the ITPP from 35.5 days (controls) to 65.5 days, similar to the effects of treatment with FOLFOX monotherapy (62 days), the standard chemotherapy for colorectal liver metastases. When FOLFOX was combined with a VEGF-neutralizing antibody, survival was prolonged to 80 days. However, when FOLFOX was combined with ITPP (10 days of treatment, with ITPP and FOLFOX five times each on alternate days), survival reached 153 days, clearly demonstrating the potentiating effects of ITPP. This influence was accompanied by a normalization of tumor-associated vasculature in histological assessment. We were also intrigued to find that survival was similarly extended (152 days) when FOLFOX was given following ITPP treatment, consistent with a long-lasting stabilization of tumor vasculature, as also evinced through a lasting downregulation of circulating angiogenesis markers.

Clinical evidence to date

Envisioning the preclinical results, the company NormOxys conducted a phase I clinical trial in unaffected volunteers (n = 36), using ITPP applied by intravenous infusion during 4 h. A dose escalation was implemented with a starting dose of 750 mg/m^2 (n = 4), and stepwise doubled (n = 4/dose) to the highest concentration of 5600 mg/m^2 (n = 4). The control group (n = 12) received dextrose only. Treatment-emerged adverse effects (TEAEs) were observed in 13 patients (9/24 in the ITPP-treated group vs 4/12 in the control group). Of these 13, ten were considered to be unrelated to the study drug, and three were considered as unlikely to be related to the study drug. All TEAEs resolved except for a mild upper respiratory infection at a dose of 3750 mg/m^2, which was not considered to be treatment related. No serious adverse effects (SAEs) or deaths associated with TEAEs were noted.

Based on these results, a dose of 5600 mg/m^2 is well tolerated in unaffected volunteers. Dosage increment in cancer patients has been approved by the U.S. Food and Drug Administration (FDA).

In 2014, a first cancer patient was treated at the University Hospital Zurich with ITPP under a compassionate use approval (Swissmedic special application Nr. SBH12-1310). Because of the first-time use in a severely ill patient, ITPP was cautiously administered as monotherapy at 5600 mg/m^2 once a week for 5 weeks. No effect (apart from an improved subjectively reported well-being of the patient) of the drug was noted, and the disease steadily progressed during the treatment time. Side effects noted were decreases in potassium (substituted orally with Kalium Effervetten® 30 mmol) and magnesium levels (substituted orally with Magnesiocard Gran® 10 mmol).

Apart from the fact that the patient had a malignancy that had proved refractory to all currently available treatments, we believe the ITPP dose applied was insufficient to achieve any anticancer effect. Measurement of the blood oxygen saturation indicated a slight increase in the p50 value, followed by a relatively rapid decline in this value following each dose of ITPP, consistent with the short half-life of ITPP (see Investigator Brochure). This pattern suggests the induction of short periods of increased oxygen pressure interspersed with a hypoxic state. Conceivably, the anticancer efficacy of ITPP is expected to depend on the installation of a continuous normoxic stage for more than a week to enable the normalization of tumor-associated vessels. Therefore, an over-cautious approach for ITPP dosing appears not to be appropriate[20].

In contrast, in our successful mouse experiments, ITPP was re-administered every second day (before oxygen saturation levels dropped back to baseline). Furthermore, the dosage we used for mice was five times higher (28,000 mg/m^2) than that given to the patient. We conclude that the success of ITPP depends strongly on a sufficiently high dose and, above all, on a sufficiently frequent administration of the drug. To avoid any potential cross reaction, we currently do not advise giving patients ITPP and standard chemotherapy simultaneously. Rather, a sequential administration would enable a higher dosing flexibility and a better control of potential side effects. Data from our syngeneic liver metastasis model indicate that ITPP effects last for at least a month following treatment cessation. These effects include normalization of vessels, inhibition of inflammation, glycolysis, and invasiveness, and promotion of beta-oxidation to levels similar to those observed immediately after treatment. These findings strongly suggest that the re-installation of tumor normoxia through ITPP is relatively stable, consistent with the normalization of tumor-associated vasculature[21]. This stability explains why ITPP can potentiate subsequent chemotherapy as efficiently as when given on alternate days during the ITPP treatment. Therefore, ITPP has the ability to create a window of therapeutic opportunity in preclinical models given sufficiently frequent administration at sufficiently high doses. The therapeutic window thus supports the use of ITPP for the potentiation of subsequent chemotherapy, minimizing potential drug interactions in patients.

General study course

This is a phase Ib dose-escalation study on the effects of ITPP in patients suffering from primary and secondary hepatic, biliary, pancreatic, or colorectal malignancies not curable by surgical means. The study design is exploratory, prospective, open-labeled, and monocentric and consists of a dose-escalation part with a maximum planned inclusion of 48 participants.

The efficacy of the study drug ITPP will be assessed before patients are administered standard chemotherapy. Patients fulfilling inclusion criteria will be treated with ITPP three times a week for 3 weeks, resulting in nine applications in total. Tumor response will be evaluated after these nine applications by positron emission tomography (PET)/CT and magnetic resonance imaging (MRI), as well as by measurement of circulatory

tumor/angiogenesis markers in serum. The anti-hypoxic activity of ITPP will be estimated through the levels of glucose uptake (PET, with the reduction in glucose uptake as a measure for the inhibition of the Warburg effect) combined with levels of tumor-specific and systemic angiogenic markers. The inhibition of hypoxia through ITPP is expected to result in a marked downregulation of pro-angiogenic serum markers, similar to what we observed in preclinical models immediately and a month after ITPP treatment. Additionally, a baseline assessment will be performed before study drug application and after subsequent chemotherapy.

After the application of ITPP, two safety precautions are planned:

- A safety period of minimum 5 and maximum 10 days between the last (normally 9th) application of ITPP and start of the standard chemotherapy to ensure a washout of ITPP and reduce the risk of possible interactions of ITPP and standard chemotherapy.
- A reduced dose of the first standard chemotherapy application to 50% of the normal dose to ensure a safe first application of standard chemotherapy after preconditioning the tumors with ITPP. If tolerated, an increase to normal doses will be performed in consequent cycles.

Dose escalation and dose rationale

The aim of the dose-escalation study is to assess the safety and tolerability data for the maximum tolerated dose (MTD). Safety is assessed according to the Common Terminology Criteria for Adverse Events (CTCAE), also known as common toxicity criteria (CTC). These criteria are issued by the US National Cancer Institute for the standardized classification of adverse effects of drugs used in cancer therapy. Specific symptoms and conditions define each level of this classification system, ranging from 1 to 5, where 1 is the mildest form of the specific symptom and 5 is death. The CTCAE version used for this study is version 4.03.

According to CTCAE, dose-limiting toxicity (DLT) will be defined as:

- any grade 5 toxicity,
- any grade 4 neutropenia lasting >7 days,
- any grade 3 febrile neutropenia, thrombocytopenia, or anemia, and
- any grade 3 or 4 nonhematologic toxicity, excluding alopecia,

that the investigators consider to be definitely, probably, or possibly related to ITPP.

A significant treatment-emergent toxicity ([s]TET) is defined as any other grade 2 toxicity considered by the investigators to be definitely, probably, or possibly related to ITPP, but not fulfilling the criteria for DLT.

Based on the assessment of DLT and TET, the MTD will be determined. MTD is defined as the dose preceding the dose level at which one patient in the cohort experiences a DLT during the treatment cycle, or the dose level at which three or more patients experience a sTET.

No more than six patients (3+3 patients) will receive the same dose during the dose-escalation phase, according to the following rules. Dose escalation to the subsequent cohort is possible if there is no DLT and no significant TET.

- If one DLT occurs, the ongoing cohort will be stopped, and another three patients will receive the next lower dose level tested, if only three patients have received this lower dose.

- If six patients already have received this lower dose, the lower dose represents the MTD.
- If DLT occurs in cohort 1, the dose will be reduced to 4200 mg/m2 weekly (i.e., to 75%), and this dose will be tested in three patients. If 1-2 significant TETs occur, another three patients will receive the same dose.
- If three or more significant TETs occur among 6 patients with the same dose, this dose level will be the MTD.
- If 1-2 of 6 patients experience significant TETs, the dose may be increased to the next dose-escalation step as shown in Table 1 in three additional patients. However, if a DLT has been observed, this dose must not be administered again.

Two factors must be considered for the ITPP dose-escalation part of this study: total dose administered and infusion rate. The human equivalent dose-corrected no observed adverse effect level (NOAEL) in rats was 15,000 mg/m^2 once a week for 5 weeks, whereas it was 43,750 mg/m^2 in minipigs, also once weekly (unpublished data obtained from NormOxys®). From animal studies, hypernatremia is associated with high plasma concentrations typically encountered at the end of the ITPP infusion and probably associated with the rate of infusion. Therefore, infusion rates of not more than 750 mg/m^2/min are recommended. According to available phase I data in healthy volunteers (unpublished data obtained from NormOxys®), cohort 1 starts at a dose close to 5600 mg/m^2 weekly, i.e., 1866 mg/m^2 three times a week. Dose escalation is performed according to the following scheme up to a maximum calculated dose of 43,700 mg/m^2 weekly, which corresponds to the NOAEL administered once weekly in minipigs. Following these rules, a maximum of 48 patients are included in the dose-escalation phase of the study. In parallel, a maximum of three patients receiving the same dose of the investigational medicinal product (IMP) will be included in the study. Depending on the safety and tolerability of the compound, the next patient (or at maximum the next three patients) who receives a higher dose will be included after the last patient completes the whole IMP cycle (with a total of nine applications), receiving the last lower dose.

We chose a classical 3+3 phase I design for the dose-escalation part of the study because information is insufficient regarding the safety and tolerability of doses above 5600 mg/m^2 once weekly and of repeated doses in humans (unpublished data obtained from NormOxys®). This design induced the least toxicity in a simulation study comparing various dose-finding escalation strategies and therefore appears to be most appropriate for a study using this substance in terminally ill patients with cancer, for whom the therapeutic benefit is unknown.

The infusion rate must not exceed 750 mg/m^2/min. To ensure this ceiling, we chose a constant infusion rate at 1/10 of the NOAEL infusion rate, so that an infusion rate of 75 mg/m^2/min was scheduled for all dose steps. Because of the short half-life of ITPP and the proposed mechanism of action, a slow, constant infusion over several hours appears more promising than a short infusion leading to excessively high concentrations of the compound.
ITPP will therefore be applied by intravenous infusion over 8 h because (i) this application mode has been safely applied in a Phase I clinical trial, and because (ii) ITPP targets the hemoglobin within erythrocytes. Intravenous application thus is the most direct way of drug delivery, lowering the dose needed to achieve biological effects. In our preclinical models, we compared intravenous, intraperitoneal, and oral application of ITPP and found that the intravenous route was the most efficient in terms of the induced shift in blood oxygen saturation.

We plan to apply ITPP three times a week, i.e., following the same regimen that we used for our preclinical studies. Injection of ITPP on this schedule enables consistent normoxia even at low doses of the compound. Following intravenous injection of ITPP into mice, oxygen dissociation curves rapidly shift to the right (indicating increased dissociation of oxygen from hemoglobin). One day after injection, the right shift drops to half and continues to decline exponentially each day, mirroring the decline in ITPP effects over time. To prevent a re-emergence of hypoxia, we re-inject ITPP on the second day, well before the right shift returns to baseline. This step is particularly important for low doses of ITPP that are expected to induce only a minor shift in oxygen saturation. Were intervals between the single injections to be longer, low doses of ITPP would not suffice to maintain normoxia. This scenario might carry the risk of inducing a state of intermittent hypoxia (periods of normoxia interrupted by periods of hypoxia), which is thought to have cancer-promoting effects that are at least comparable to those of persistent hypoxia. In other published preclinical studies, ITPP was applied once a week. However, in these studies, the ITPP dose was 28,000 mg/m^2 or higher (i.e., ≥5 times higher than the highest dose tested in humans), an amount sufficient to maintain normoxia over a week. Because we have to start dose increment with a safe dose (5600 mg/m^2), we will inject ITPP frequently enough to avoid any potential issues associated with alternating levels of tissue oxygen.

ITPP will be given nine times over a period of 3 weeks. The arguably most desired effect of ITPP is the normalization of tumor vasculature. In mice, we could achieve this within 10 days (five injections, one on every second day). Currently, no data are available to estimate the time needed to achieve vessel normalization through ITPP in human vessels. To consider a potentially longer period in humans, we have arbitrarily chosen 3 weeks for patients. Following this period, levels of circulating angiogenic markers will be measured and compared to pre-treatment levels.

Dose finding, including the MTD, is the primary objective of this study. We will start with the highest tested dose that was found to be safe in healthy volunteers and in one patient with end-stage cancer (5600 mg/m^2). A dose of 28,000 mg/m^2 was demonstrated to be efficient in animal cancer models, and a dose of 5600 mg/m^2 had limited anticancer effects in either the preclinical models or the single patient with cancer. The planned maximum dose is 43,700 mg/m^2, which is the dose that has been safely tested in minipigs (43,750 mg/m^2). According to our calculations, the first mild cytotoxicity from ITPP would be expected at around 900,000 mg/m^2, a dose that is about 20 times higher than our planned maximum dose.

Risk–benefit considerations

ITPP is the first anti-hypoxic drug with a great potential for clinical use. Its anti-hypoxic action not only demonstrates anti-cancer effects in preclinical models but also is expected to lead to the normalization of tumor-associated vessels. As a consequence, ITPP may improve the efficacy of subsequent standard chemotherapy and thus could be integrated into existing treatment schedules for patients with cancer. Our observations suggest that the anti-hypoxic effects of ITPP are relatively long-lasting (at least a month following cessation), providing a generous time window for a subsequent treatment. Therefore, the main expectation is that ITPP might be of benefit for enhancing the efficacy of subsequent chemotherapy.

From all what we know thus far, ITPP is characterized by a very low intrinsic toxicity. One group tested the cytotoxicity of ITPP in cell cultures and observed minor toxicity only at the highest dose tested. Of note, this ITPP dose was much higher (8 times) than the calculated values in peripheral tissues upon intravenous application of the doses proposed for this study. For context, most conventional chemotherapies demonstrate significant cytotoxicity at doses used in cancer patients. No preclinical cancer study on ITPP published to date has reported any signs of toxicity associated with this novel compound.

Considering the anticancer effects together with the low toxicity of ITPP, we believe that the potential benefits clearly outweigh the potential risks associated with the human use of this compound. The study is designed to start with a dose that was safely tested in humans, followed by a careful increment of dosing with each patient group. This approach will allow for strict control of any side effects that emerge.

Assuming the lack of significant side effects, the worst case would be the absence of anti-cancer effects. Given that all patients will subsequently receive the current standard treatment, such a worst-case scenario would not negatively affect patient management. Even in such a case, ITPP still might exert some beneficial effects through improved provision of oxygen. In animals, improved oxygenation has, for example, been shown to boost physical performance capacity. In patients with cancer, improved oxygenation may lead to a better general state, contributing to an improved quality of life.

As noted, ITPP is the first anti-hypoxic drug available for human use. Vascular normalization and the associated improved oxygenation might enhance the effects of chemotherapy and other compounds delivered intravenously, along with radiotherapy. Given that hypoxia occurs in virtually any solid tumor beyond a certain size, the success of this trial might have a considerable impact on future treatment strategies for many cancer types. As such, we consider the knowledge gained from our endpoints to be of general importance for the future management of patients with cancer.

Study objectives

Overall objective

The purpose of this study is to evaluate whether the novel anti-cancer drug ITPP is safe and tolerated in patients with primary and secondary hepato-pancreato-biliary neoplasia as measured by exploring the MTD.

Primary objective

The primary study aim is to determine the safety and tolerability of ITPP in patients with primary and secondary hepato-pancreato-biliary neoplasia, as measured by exploring the MTD in a conservative 3+3 dose-escalation schedule. The window for DLT assessment is from the first dose of the study drug to the first dose of standard of care chemotherapy or at 10 days following completion of the last dose of the study drug (whichever is shorter in duration). Specifically, the study aim is to determine:

- the safety and tolerability of ITPP in the treatment of patients with hepato-pancreatico-biliary tumors, and
- the MTD of ITPP in patients with hepato-pancreatico-biliary tumors by interpatient dose escalation.

Secondary objectives

The secondary objectives of this study are to assess the anticancer efficacy of ITPP on decreasing tumor volume, metabolic activity, and circulatory tumor and angiogenic markers. Specifically, we seek to:

- assess the plasma pharmacokinetics of intravenous doses of ITPP, and
- determine the biochemical and radiographic antitumor efficacy of ITPP monotherapy and consequent chemotherapy in patients with hepato-pancreatico-biliary tumors.

Safety objectives

The study aims to assess the safety of ITPP and its tolerability in terms of incidence of treatment-emergent toxicities and especially electrolyte changes. Monitoring of the central nervous, respiratory, and renal systems is in place and will be assessed. Occurrences of adverse events (AEs) will be carefully monitored during the study.

Study outcomes

Primary outcome

Assessment of (i) safety and patient tolerance of increasing doses of ITPP, and (ii) establishment of the MTD (primary endpoint) according to the dose escalation schema s measured by collection of adverse effect information as measured by collection of adverse effects information according to Common Terminology Criteria for Adverse Events (CTCAE, US National Cancer Institute, version 4.03).

Secondary outcomes

The pharmacokinetics of increasing doses of ITPP administration will be measured using repeated blood measurements (plasma). Pharmacokinetic parameters assessed include (i) area under the curve (AUC) of the concentration versus time curve from time zero to the last measurable concentration, (ii) AUC from time zero extrapolated to infinity maximum observed concentration (C_{max}), (iii) time of observed C_{max}, (iv) trough serum concentration, (v) total body clearance, (vi) terminal elimination half-life ($t_{1/2}$), and (vii) volume of distribution at steady state.

The efficacy of ITPP monotherapy and subsequent chemotherapy will be measured by radiological assessment, with imaging to be performed before and after ITPP therapy, as well as after chemotherapy, if possible, using fluorodeoxyglucose F18 PET (response evaluated by European Organisation for Research and Treatment of Cancer criteria[22]) and MRI (response evaluated by RECIST 1.1[23]); and by biochemical assessment, using measurement of specific tumor and angiogenesis markers in serum.

Safety outcomes

The safety and tolerability of ITPP is the primary outcome of our study and will be carefully addressed with regard to AEs and adverse reactions.

Study population

Patients with primary and secondary hepato-pancreato-biliary neoplasia will be assessed for eligibility at the interdisciplinary tumor board of the Swiss HPB Center of the University Hospital Zurich. Patient information and informed consent will be obtained in both the outpatient and inpatient hospital settings. Prior to study inclusion, all cases will be discussed at the institutional multidisciplinary tumor board. Overall, a maximum of 48 patients will be included in the dose escalation. Drug application will be performed in an outpatient setting in the Phase I unit of the Clinical Trials Centers of the University Hospital Zurich where no competing studies are planned, and the study equipment is explicitly reserved for this study, including appropriate backup in case of technical malfunction. Study drug preparation (vials) will be provided by Carbogen Amcis AG (Aarau, CH) and Baccinex SA (Courroux, CH) and stored at Cantonal Pharmacy Zurich (KAZ). KAZ is responsible for the sterile production of the final study IMP (infusion according to patient weight). The study is planned to be completed within a maximum of 3 years.

Inclusion criteria

Participants fulfilling all of the following inclusion criteria are eligible for the study:

- Signed informed consent as documented by signature after being informed about the study drug, aims of the study, and potential risks and benefits of participation in a language understandable to the patient and a minimal time for consideration of 24 h
- Patient diagnosed with non-resectable hepato-pancreato-biliary neoplasm
- Estimated life expectancy >3 months
- Male and female patients ≥18 years of age
- Eastern Cooperative Oncology Group performance status score ≤1 at study entry
- Adequate hematologic function, as defined by:
    - absolute neutrophil count ≥1.00 G/L
    - hemoglobin level ≥100 G/L
    - platelet count ≥100 G/L
- Adequate renal function, as defined by a serum creatinine ≤160 µmol.
- Adequate hepatic function, as defined by:
    - aspartate transferase and alanine transferase ≤ 125 U/L (in case of known liver involvement, ≤250 U/L)
    - bilirubin ≤38 umol/L
- Adequate recovery from recent surgery, chemotherapy, and radiation therapy; at least 28 days must have elapsed since major surgery, prior chemotherapy, prior treatment with an investigational agent or device, or prior radiation therapy (palliative radiation therapy is allowed)
- Accessible for treatment and follow-up

Exclusion criteria

The presence of any one of the following exclusion criteria will lead to exclusion of the participant for study participation:

- Contraindications to the class of drugs under study, e.g., known hypersensitivity or allergy to class of drugs or the investigational product
- Women who are pregnant or breast feeding
- Intention to become pregnant during the course of the study
- Lack of safe contraception, defined as: female participants of childbearing potential, not using and not willing to continue using a medically reliable method of contraception for the entire study duration, such as oral, injectable, or implantable contraceptives, or intrauterine contraceptive devices, or who are not using any other method considered sufficiently reliable by the investigator in individual cases. Please note that female participants who are surgically sterilized/hysterectomized or post-menopausal for longer than 2 years are not considered as being of child-bearing potential.
- Other clinically significant concomitant disease states (e.g., renal failure, hepatic dysfunction, cardiovascular disease, etc.)
- Known or suspected non-adherence or drug or alcohol abuse
- Inability to follow the procedures of the study, e.g., because of language problems, psychological disorders, dementia, etc.
- Participation in another study with an investigational drug within the 30 days preceding and during the present study
- Previous enrollment in the current study
- Enrollment of the investigator, investigator family members, employees, and other dependent persons

Justification for choice of study population

The choice of the study population and the rationale for it is based on results in preclinical animal models of cancer diseases. No vulnerable participants (e.g., minors, participants incapable of judgment, participants under tutelage, emergency situations) will be included.

Recruitment and screening

All consecutive candidates diagnosed with primary and secondary hepato-pancreato-biliary neoplasia will be assessed for eligibility at the interdisciplinary tumor board of the Swiss HPB Center of the University Hospital Zurich. An investigator physician will inform patients about the study orally and in writing. Patients who are willing to participate, providing written informed consent, and fulfilling the inclusion/exclusion criteria, will be included into the study. Enough time will be provided to patients to decide whether to participate (or not) in the trial (i.e., at least 24 h).

Criteria for withdrawal/discontinuation of participants

Scenarios under which a participant must be withdrawn from the study prior to the expected completion include:

- safety reasons: non-correctable electrolyte changes;
- failure of participant to adhere to protocol requirements; and
- participant consent withdrawal.

If abrupt termination of study treatment could affect participant safety, the patient will be carefully monitored, if necessary, in an inpatient setting. An interdisciplinary decision will be taken about whether to proceed with study drug or to exclude the participant. Participants who discontinue the study will be replaced according to the 3+3 escalation schedule. Participant adherence with the study procedures is expected to be high because the study drug is applied intravenously in an inpatient hospital setting.

Time windows for study visits include patient information and informed consent followed by initial patient assessment in an inpatient setting. Afterwards, ITPP application (three times per week over 3 weeks) in the phase I unit of the CTC is planned. Within 10 days after the last ITPP application, a reassessment will be performed followed by standard chemotherapy.

A priori, an interval

- of >7 days between two ITPP applications leads to a discontinuation of the participant with a replacement by another patient; or
- of <5 IMP applications within 5 weeks leads to a discontinuation of the participant with a replacement by another patient.

Based on this study schedule, a patient will be enrolled in the study a minimum of 4 weeks and a maximum of 9 weeks. The procedure after missed appointments will be discussed in the interdisciplinary team for each patient, with a decision made about whether to continue with the ITPP treatment or a discontinuation of the participant is indicated with a replacement by another patient. It is important to note that each patient will be informed that a withdrawal has no influence on the patient's further standard treatment.

Experimental intervention

The investigational product ITPP (brand name: OXY111A), which exists in stable form as a hexasodium salt (ITPP/Na6), will be prepared in three steps, as done in the phase Ia trial with healthy volunteers:

- Production of solid compound (by Carbogen Amics AG, Aarau, Canton Aargau, Switzerland): ITPP existing as white powder.
- Production of vials (by Baccinex SA, Courroux, Canton Jura, Switzerland): ITPP existing as translucent solution; composition: 10 mL vial containing 375 mg/mL ITPP. The study drug will then be shipped refrigerated in boxes of 100 vials to the Pharmaceutical Department of KAZ. At KAZ, they will be stored under refrigerated controlled settings (range, 2–8° C, no direct sunlight).
- Production of patient-specific sterile infusions by KAZ. Because of its anionic properties, ITPP is a potent chelator of divalent cations and has been shown to cause hypocalcemia in preclinical models. Therefore, ITPP administration with Ca++-chloride (Baxter) at a 1:0.75 molar ratio has been determined to prevent hypocalcemia. The respective doses of ITPP and CaCl2 are calculated based on body surface area (calculated using the DuBois/DuBois formula) and admixed in a total volume of 1030 mL dextrose 5 solution on the same day or at the earliest on the evening before and stored at 4°C until administration.
- Full details for the preparation of the test product and reference therapy administration, including the rate of infusion, total infusion volume for each dose level, and admixing with calcium chloride for the test product only (ITPP injection) are provided in the separate pharmacy manual (detailed admixing procedure on page 6 and following).

In summary, appropriate volumes of ITPP (375 mg/mL) and calcium chloride (5.5%) are calculated based on patient surface area and the formulas provided. Carrier infusion is 5% dextrose (D5W). The amount to be removed from a 1000-mL bag is then calculated. This volume of D5W is removed from the infusion bag so that the total volume after addition of ITPP and calcium chloride will be 1030 mL. ITPP solution is added and mixed thoroughly with the D5W. Calcium chloride solution is added rapidly, 10 mL or less at a time. The bag contents are mixed vigorously after each addition. Rapid addition of calcium chloride with immediate mixing is crucial to ensure that locally high concentrations of calcium chloride do not persist in contact with ITPP. Persistence of locally high calcium chloride concentrations in the presence of ITPP has been shown to cause precipitation.

The sterile infusion will be preferably applied through a central vein catheter (Port a Cath System) if available; otherwise, it will be applied through a peripheral venous catheter under controlled settings via an intravenous pump.

Regular study drug reconciliation checks will be performed to document drug assigned, drug consumed, and drug remaining (i.e., how much drug was assigned and whether participants actually received the assigned dose or received the dose properly, how much remains, how much drug was inadvertently damaged). This reconciliation will be logged on the drug accountability form and signed and dated by the study team, recorded by KAZ, and added to the eCRF.

Safety

During the entire duration of the study, all adverse events (AEs) and all serious AEs (SAEs) are collected, fully investigated and documented in source documents and electronic case report forms (eCRF). Study duration encompasses the time from when the participant signs the informed consent until the last protocol-specific procedure has been completed, including a safety follow-up period.

Definition of (S)AEs and other safety-related events

AEs

AEs are defined as any untoward medical occurrence in a patient or clinical investigation participant administered a pharmaceutical product and which does not necessarily have a causal relationship with this treatment. An AE can therefore be any unfavorable and unintended sign (including an abnormal laboratory finding), symptom, or disease temporally associated with the use of a medicinal study product, whether or not it is related to the medicinal study product.

An AE may also consist of a new disease, an exacerbation of a pre-existing illness or condition, a recurrence of an intermittent illness or condition, a set of related signs or symptoms, or a single sign or symptom.

AEs observed by the investigator and/or reported by the participant must be reported in the eCRF during the entire study period, i.e., the period of time from the first engagement (signature of informed consent) to the last protocol-specific procedure, regardless of the medicinal study product relation assessment.

For all AEs, sufficient information will be pursued and/or obtained to permit an adequate determination of the outcome of the event (i.e., whether the event should be classified as an SAE) and an assessment of the causal relationship between the AE and the investigational drug or study treatment(s).

Whenever available, the underlying disease or condition for which a therapeutic or diagnostic procedure is required should be reported as the AE term. Surgeries or other invasive procedures that had already been planned prior to the start of the study do not have to be documented as AEs. These planned procedures will be recorded in the eCRF by the investigator at the baseline visit. It is not important if the condition was known before enrollment, only if the procedure was planned before.

SAEs

SAEs comprise any untoward medical occurrence that at any dose:

- results in death,
- is life-threatening,
- requires participant hospitalization or prolongation of current hospitalization,
- results in persistent or significant disability/incapacity, or
- results in a congenital anomaly/birth defect, or
- any important medical event and any event which, though not included in the above, may jeopardize the participant or require intervention to prevent one of the outcomes listed above.

Any other medically important condition that may be not immediately life-threatening or results in death or hospitalization but may jeopardize the participant or may require intervention to prevent one of the outcomes listed above should also usually (i.e., based on medical and scientific judgment) be considered serious. For example:

intensive treatment at home for allergic bronchospasm; certain laboratory abnormalities (e.g., blood dyscrasias); convulsions that do not result in hospitalization; and development of drug dependency or drug abuse.
SAEs should be followed until resolution or stabilization. Participants with ongoing SAEs at study termination (including safety visit) will be further followed up until recovery or until stabilization of the disease after termination.

Unexpected adverse drug reaction
An "unexpected" adverse drug reaction is an adverse reaction, the nature or severity of which is not consistent with the applicable product information (e.g., Investigator's Brochure for drugs that are not yet approved and Product Information for approved drugs, respectively).

Suspected unexpected serious adverse reaction (SUSAR)
A serious adverse reaction, the nature or severity of which is suspected to be inconsistent with the applicable product information.

Recording of (S)AEs and other safety-related events
Clinical investigators and ultimately the principal investigator (PI) have the primary responsibility for AE identification, documentation, grading, and assignment of attribution to the investigational agent/intervention.
Clinical study participants will be routinely questioned about AEs at study visits. The well-being of the participants will be ascertained by neutral questioning ("How are you?"). The investigator is responsible for reporting all AEs occurring during the course of the study.
All observed or volunteered adverse drug events (serious or non-serious) and abnormal test findings, regardless of treatment group or suspected causal relationship to the investigational drug or study treatment(s), will be recorded in the patient file and subsequently in the eCRF.
AEs or abnormal test findings felt to be associated with the study treatment(s) will be followed until the event (or its sequelae) or the abnormal test finding resolves or stabilizes at a level acceptable to the investigator. An abnormal test finding will be classified as an AE if one or more of the following criteria are met:

- The test finding is accompanied by clinical symptoms.
- The test finding necessitates additional diagnostic evaluation(s) or medical/surgical intervention; including significant additional concomitant drug treatment or other therapy
    - **Note:** simply repeating a test finding, in the absence of any of the other listed criteria, does not constitute an AE.
- The test finding leads to a change in study dosing or discontinuation of participant participation in the clinical study.

All AEs, serious and non-serious, will be fully documented in the appropriate eCRF. For each AE, the investigator will provide the onset, duration, intensity, treatment required, outcome, and action taken with the investigational product.

Assessment of (S)AEs and other safety-related Events

The investigator will promptly review documented AEs and abnormal test findings to determine if:

- the abnormal test finding should be classified as an AE,
- there is a reasonable possibility that the AE was caused by the investigational drug or study treatment(s), and
- the AE meets the criteria for an SAE.

The intensity of an AE will be assessed by the investigator as:

- mild (hardly noticeable, negligible impairment of well-being),
- moderate (marked discomfort, but tolerable without immediate relief), or
- severe (overwhelming discomfort, calling for immediate relief).

The investigator's assessment of causality by the study drug is done according to the following definitions:

Unrelated	An AE that<br>• shows no temporal relationship to drug administration, and/or<br>• other drugs or chemicals or underlying disease provide definite explanations.
Unlikely	An AE with a<br>• temporal relationship to drug administration that makes a causal relationship improbable, and/or<br>• other drugs or chemicals or underlying disease provide plausible explanations.
Possible	An AE that<br>• occurs within a reasonable time sequence to administration of the drug, but<br>• could also be explained by concurrent disease or other drugs or chemicals.
Probable	An AE that<br>• occurs within a reasonable time sequence to administration of the drug, and/or<br>• is unlikely to be attributed to concurrent disease or other drugs or chemicals.
Definite	An AE that<br>• occurs within a highly plausible time relationship to IMP administration, and/or<br>• cannot be explained by concurrent disease or other drugs or chemicals.

Reporting of (S)AEs and other safety-related Events

The investigator is responsible for SAE reporting to the CEC according to the following details:

- Reporting to the CEC any SAE that resulted in death:
  - without delay, and no later than 7 calendar days.
- Reporting to the CEC any fatal SAEs if evaluated as “suspected,” “unexpected,” and “drug-related” (SUSAR),
  - without delay and no later than 7 calendar days following awareness that event meets criteria for a SUSAR.
- Reporting to the CEC any nonfatal SAEs if evaluated as “suspected,” “unexpected,” and “drug-related” (SUSAR):
  - promptly and no later than 15 calendar days following awareness that the event meets the criteria for a SUSAR.
- All other SAEs will be summarized in the annual safety update report.

The Sponsor is responsible for SAE reporting to Swissmedic according to the following details:

- Compliance with the regulatory requirements of Swissmedic regarding prompt reporting of unexpected SAEs for which a causal relationship with the study drug cannot be ruled out.
- Reporting to Swissmedic of fatal SAEs if evaluated as “suspected,” “unexpected,” and “drug-related” (SUSAR):
  - without delay and no later than 7 calendar days following awareness that the event meets the criteria for a SUSAR.
- Reporting to Swissmedic of nonfatal SAEs if evaluated as “suspected,” “unexpected,” and “drug-related” (SUSARs):
  - promptly and no later than 15 calendar days following awareness that the event meets the criteria for a SUSAR.
- Sending annual safety reports, starting one year after the date of notification to Swissmedic. These reports should contain:
  - a concise critical summary of the safety profile of the drug studied as well as the safety issues that have arisen;
  - a listing of all SUSARs that have occurred in Switzerland and at the international level (if applicable); and
  - ideally all adverse drug reactions at the international level.
  - The accompanying letter provided with the annual safety report should contain a short summary of the status of the clinical trial in Switzerland (number of centers open/closed, number of patients recruited/recruitment closed, and number of SAR/SUSAR).

Statistical methods

Hypothesis: Not applicable for phase Ib dose-escalation study.

Determination of Sample Size

As usual in dose-escalation studies in early drug development, the sample size is not calculated according to statistical acceptance criteria, but based on dose-escalation rules as described above. Sample size was therefore based on the 3+3 rule for dose escalation with eight dose levels, which corresponds to a maximum of 48 patients. No more than six patients (3+3 patients) will receive the same dose during the dose-escalation phase.

Statistical Criteria for Termination of Trial

Not applicable; termination of this trial and the stopping rules are based on the safety parameters rather than on statistical criteria.

Primary Analysis

The primary analysis will take place directly after definition of the MTD of IMP. The primary analysis focuses on safety parameters. Participants reporting any AEs, the occurrence of specific AEs, and discontinuation because of AEs will be summarized using descriptive statistics. The sample size depends on the three-patient cohort study design to provide a safety stopping rule in the event of a DLT.

Secondary Analyses

For analysis of the efficacy data, we will calculate mean values and standard deviations. Assuming a first-order rate equation and thus a non-Guassian distribution of efficacy values, we will computed statistical significance using non-parametrical testing for repeated measurements (Kruskal–Wallis test).

Interim Analyses

After each cohort, an analysis of the gathered data is planned.

Safety Analysis

Analysis of safety parameters will be done after each single IMP application as well as after completion of the whole IMP cycle for the patient's individual safety as well as for decision-making for next higher dose level or defining MTD, respectively. Safety parameters will be analyzed primarily by a physician investigator and discussed within the interdisciplinary study team. The PI is responsible for the final decision regarding further procedures.

Deviation(s) from the Original Statistical Plan: No deviation(s) from the planned analyses will be justified. Any deviation will be reported at the study end report.

Handling of Missing Data and Drop-Outs: If safety assessment cannot be completed for a complete cycle, the patient will be excluded from the analysis and replaced by another patient in the same cohort to guarantee a minimum of three patients per cohort before escalation to the next cohort.

R Session information for statistical analyses

R version 4.0.2 (2020-06-22)

Platform: x86_64-w64-mingw32/x64 (64-bit)

Running under: Windows 10 x64 (build 19042)

Matrix products: default

locale:

[1] LC_COLLATE=German_Switzerland.1252 LC_CTYPE=German_Switzerland.1252 LC_MONETARY=German_Switzerland.1252

[4] LC_NUMERIC=C LC_TIME=German_Switzerland.1252

attached base packages:

[1] grid stats graphics grDevices utils datasets methods base

other attached packages:

[1] survminer_0.4.9 pkr_0.1.2 rtf_0.4-14.1 forestplot_1.10.1 checkmate_2.0.0 magrittr_2.0.1

[7] binr_1.1 foreign_0.8-80 rms_6.2-0 SparseM_1.81 Hmisc_4.5-0 Formula_1.2-4

[13] survival_3.2-11 lattice_0.20-41 corrplot_0.84 scales_1.1.1 ggsignif_0.6.1 rJava_1.0-4

[19] ggsci_2.9 gridExtra_2.3 ggpubr_0.4.0 readxl_1.3.1 forcats_0.5.1 stringr_1.4.0

[25] dplyr_1.0.5 purrr_0.3.4 readr_1.4.0 tidyr_1.1.3 tibble_3.1.1 ggplot2_3.3.3

[31] tidyverse_1.3.1

loaded via a namespace (and not attached):

[1] TH.data_1.0-10 colorspace_2.0-0 ellipsis_0.3.2 rio_0.5.26 rsconnect_0.8.17 htmlTable_2.1.0

[7] base64enc_0.1-3 fs_1.5.0 rstudioapi_0.13 farver_2.1.0 MatrixModels_0.5-0 fansi_0.4.2

[13] mvtnorm_1.1-1 lubridate_1.7.10 xml2_1.3.2 R.methodsS3_1.8.1 codetools_0.2-16 splines_4.0.2

[19] knitr_1.33 jsonlite_1.7.2 km.ci_0.5-2 broom_0.7.6 cluster_2.1.0 dbplyr_2.1.1

[25] R.oo_1.24.0 png_0.1-7 compiler_4.0.2 httr_1.4.2 backports_1.2.1 assertthat_0.2.1

[31] Matrix_1.2-18 cli_2.5.0 htmltools_0.5.1.1 quantreg_5.85 tools_4.0.2 gtable_0.3.0

[37] glue_1.4.2 Rcpp_1.0.6 carData_3.0-4 cellranger_1.1.0 vctrs_0.3.8 nlme_3.1-148

[43] conquer_1.0.2 xfun_0.22 openxlsx_4.2.3 rvest_1.0.0 lifecycle_1.0.0 rstatix_0.7.0

[49] polspline_1.1.19 MASS_7.3-53.1 zoo_1.8-9 hms_1.0.0 sandwich_3.0-0 RColorBrewer_1.1-2

[55] yaml_2.2.1 curl_4.3.1 KMsurv_0.1-5 rpart_4.1-15 latticeExtra_0.6-29 stringi_1.5.3

[61] highr_0.9 zip_2.1.1 rlang_0.4.11 pkgconfig_2.0.3 matrixStats_0.58.0 evaluate_0.14

[67] labeling_0.4.2 htmlwidgets_1.5.3 tidyselect_1.1.1 R6_2.5.0 generics_0.1.0 multcomp_1.4-17

[73] DBI_1.1.1 pillar_1.6.0 haven_2.4.1 withr_2.4.2 abind_1.4-5 nnet_7.3-14

[79] modelr_0.1.8 crayon_1.4.1 car_3.0-10 survMisc_0.5.5 utf8_1.2.1 rmarkdown_2.7

[85] jpeg_0.1-8.1 data.table_1.14.0 reprex_2.0.0 digest_0.6.27 xtable_1.8-4 munsell_0.5.0

[91] viridisLite_0.4.0

Supplementary references

1. Limani P, *et al.* Development of OXY111A, a novel hypoxia-modifier as a potential antitumor agent in patients with hepato-pancreato-biliary neoplasms - Protocol of a first Ib/IIa clinical trial. *BMC Cancer* **16**, 812 (2016).
2. Kerbel RS. Tumor angiogenesis. *N Engl J Med* **358**, 2039-2049 (2008).
3. Potente M, Gerhardt H, Carmeliet P. Basic and therapeutic aspects of angiogenesis. *Cell* **146**, 873-887 (2011).
4. Bao B, *et al.* The biological kinship of hypoxia with CSC and EMT and their relationship with deregulated expression of miRNAs and tumor aggressiveness. *Biochim Biophys Acta* **1826**, 272-296 (2012).
5. De Bock K, Mazzone M, Carmeliet P. Antiangiogenic therapy, hypoxia, and metastasis: risky liaisons, or not? *Nature reviews Clinical oncology* **8**, 393-404 (2011).
6. Gale DP, Maxwell PH. The role of HIF in immunity. *Int J Biochem Cell Biol* **42**, 486-494 (2010).
7. Fylaktakidou KC, Lehn J-M, Greferath R, Nicolau C. Inositol tripyrophosphate: a new membrane permeant allosteric effector of haemoglobin. *Bioorg Med Chem Lett* **15**, 1605-1608 (2005).
8. Duarte CD, Greferath R, Nicolau C, Lehn JM. myo-Inositol trispyrophosphate: a novel allosteric effector of hemoglobin with high permeation selectivity across the red blood cell plasma membrane. *Chembiochem* **11**, 2543-2548 (2010).
9. Vincent SP, Lehn JM, Lazarte J, Nicolau C. Transport of the highly charged myo-inositol hexakisphosphate molecule across the red blood cell membrane: a phase transfer and biological study. *Bioorganic & medicinal chemistry* **10**, 2825-2834 (2002).
10. Aprahamian M, *et al.* Myo-InositolTrisPyroPhosphate treatment leads to HIF-1α suppression and eradication of early hepatoma tumors in rats. *Chembiochem* **12**, 777-783 (2011).
11. Biolo A, *et al.* Enhanced exercise capacity in mice with severe heart failure treated with an allosteric effector of hemoglobin, myo-inositol trispyrophosphate. *Proc Natl Acad Sci U S A* **106**, 1926-1929 (2009).
12. Derbal-Wolfrom L, *et al.* Increasing the oxygen load by treatment with myo-inositol trispyrophosphate reduces growth of colon cancer and modulates the intestine homeobox gene Cdx2. *Oncogene* **32**, 4313-4318 (2013).
13. Kieda C, Greferath R, Crola da Silva C, Fylaktakidou KC, Lehn J-M, Nicolau C. Suppression of hypoxia-induced HIF-1alpha and of angiogenesis in endothelial cells by myo-inositol trispyrophosphate-treated erythrocytes. *Proc Natl Acad Sci U S A* **103**, 15576-15581 (2006).
14. Kieda C, *et al.* Stable tumor vessel normalization with pO_2 increase and endothelial PTEN activation by inositol trispyrophosphate brings novel tumor treatment. *J Mol Med (Berl)* **91**, 883-899 (2013).
15. Raykov Z, *et al.* Myo-inositol trispyrophosphate-mediated hypoxia reversion controls pancreatic cancer in rodents and enhances gemcitabine efficacy. *Int J Cancer* **134**, 2572-2582 (2014).
16. Limani P, *et al.* Antihypoxic Potentiation of Standard Therapy for Experimental Colorectal Liver Metastasis through Myo-Inositol Trispyrophosphate. *Clin Cancer Res* **22**, 5887-5897 (2016).
17. Limani P, *et al.* The Allosteric Hemoglobin Effector ITPP Inhibits Metastatic Colon Cancer in Mice. *Annals of surgery* **266**, 746-753 (2017).
18. Sihn G, *et al.* Anti-angiogenic properties of myo-inositol trispyrophosphate in ovo and growth reduction of implanted glioma. *FEBS Lett* **581**, 962-966 (2007).
19. Wong AS, Ho EN, Wan TS. Detection of myo-inositol trispyrophosphate in equine urine and plasma by hydrophillic interaction chromatography-tandem mass spectrometry. *Drug testing and analysis* **4**, 355-361 (2012).
20. Dewhirst MW. Intermittent hypoxia furthers the rationale for hypoxia-inducible factor-1 targeting. *Cancer Res* **67**, 854-855 (2007).
21. Carmeliet P, Jain RK. Principles and mechanisms of vessel normalization for cancer and other angiogenic diseases. *Nature reviews Drug discovery* **10**, 417-427 (2011).
22. Eisenhauer EA, *et al.* New response evaluation criteria in solid tumours: revised RECIST guideline (version 1.1). *European journal of cancer* **45**, 228-247 (2009).
23. Young H, *et al.* Measurement of clinical and subclinical tumour response using [18F]-fluorodeoxyglucose and positron emission tomography: review and 1999 EORTC recommendations. European Organization for Research and Treatment of Cancer (EORTC) PET Study Group. *European journal of cancer* **35**, 1773-1782 (1999).
