# Supplementary material for: Phase Ib dose-escalation study of the hypoxia-modifier Myo-inositol trispyrophosphate in patients with hepatopancreatobiliary tumors
Source: Nat Commun. 2021 Jun 21;12:3807. doi: 10.1038/s41467-021-24069-w (PMC8217170; doi:10.1038/s41467-021-24069-w)
Supplement: Supplementary file 3 — Description of Additional Supplementary Files [file 41467_2021_24069_MOESM3_ESM.pdf]

Description of Additional Supplementary Files

Supplementary Software:

The manuscript is accompanied by a “Source Data” Microsoft excel file (NCOMMS_20_42537_SourceDataFile.xlsx) and a “Source Code” *R* markdown file (NCOMMS_20_42537_SourceCode.Rmd), which allow reproduction of all statistical analyses, calculations and graphical representations performed for the study.

R (preferred version ≥ 4.0.2, https://www.r-project.org/) and ideally *R* Studio (preferred version ≥ V1.3.1093, https://www.rstudio.com/), both freely available, should be installed for executing of the code file. The following packages are needed for the analyses: "tidyverse", "readxl", "ggpubr", "gridExtra", "ggsci", "rJava", "ggsignif", "scales", "corrplot", "rms", "pkr", "survival", "survminer", "markdown", "xfun", "knitr", "kableExtra", "tinytex". The setup section of the Rmd file will automatically check if the necessary packages are installed, install missing ones, and load them for further analyses.

To run the statistical analyses, ideally create a new *R* project inside *R* studio. Save the source data file and the source code in the main project folder/working directory and open the Rmd file. The code will automatically take you step-by-step with explanations through the analyses. It will automatically generate an output folder and save all figures as pdf in the identical resolution and size as used in the paper. The whole document can be knitted to html or pdf for future inspection.

The file includes analyses on the following aspects:

- Baseline characteristics
- Dose Escalation
- Safety, tolerability & adverse events
- Pharmacokinetics
- Radiological responses
- Biochemical responses: Tumor markers
- Biochemical responses: VEGFA, Angiopoietin 1/ANG1, Angiopoietin 2/ANG2, Epidermal Growth Factor/EGF, PECAM-1/CD31
- Correlation plot
- Survival analyses
- Histological Analyses

For further questions or enquiries, please contact the corresponding authors:
clavien@access.uzh.ch, perparim.limani@usz.ch

R session information were the following:

R version 4.0.2 (2020-06-22)
Platform: x86_64-w64-mingw32/x64 (64-bit)
Running under: Windows 10 x64 (build 19042)
Matrix products: default

locale:
[1] LC_COLLATE=German_Switzerland.1252 LC_CTYPE=German_Switzerland.1252 LC_MONETARY=German_Switzerland.1252
[4] LC_NUMERIC=C LC_TIME=German_Switzerland.1252

attached base packages:
[1] grid stats graphics grDevices utils datasets methods base

other attached packages:
[1] survminer_0.4.9 pkr_0.1.2 rtf_0.4-14.1 forestplot_1.10.1 checkmate_2.0.0 magrittr_2.0.1
[7] binr_1.1 foreign_0.8-80 rms_6.2-0 SparseM_1.81 Hmisc_4.5-0 Formula_1.2-4
[13] survival_3.2-11 lattice_0.20-41 corrplot_0.84 scales_1.1.1 ggsignif_0.6.1 rJava_1.0-4
[19] ggsci_2.9 gridExtra_2.3 ggpubr_0.4.0 readxl_1.3.1 forcats_0.5.1 stringr_1.4.0
[25] dplyr_1.0.5 purrr_0.3.4 readr_1.4.0 tidyr_1.1.3 tibble_3.1.1 ggplot2_3.3.3
[31] tidyverse_1.3.1

loaded via a namespace (and not attached):
[1] TH.data_1.0-10 colorspace_2.0-0 ellipsis_0.3.2 rio_0.5.26 rsconnect_0.8.17 htmlTable_2.1.0
[7] base64enc_0.1-3 fs_1.5.0 rstudioapi_0.13 farver_2.1.0 MatrixModels_0.5-0 fansi_0.4.2
[13] mvtnorm_1.1-1 lubridate_1.7.10 xml2_1.3.2 R.methodsS3_1.8.1 codetools_0.2-16 splines_4.0.2
[19] knitr_1.33 jsonlite_1.7.2 km.ci_0.5-2 broom_0.7.6 cluster_2.1.0 dbplyr_2.1.1
[25] R.oo_1.24.0 png_0.1-7 compiler_4.0.2 httr_1.4.2 backports_1.2.1 assertthat_0.2.1
[31] Matrix_1.2-18 cli_2.5.0 htmltools_0.5.1.1 quantreg_5.85 tools_4.0.2 gtable_0.3.0
[37] glue_1.4.2 Rcpp_1.0.6 carData_3.0-4 cellranger_1.1.0 vctrs_0.3.8 nlme_3.1-148
[43] conquer_1.0.2 xfun_0.22 openxlsx_4.2.3 rvest_1.0.0 lifecycle_1.0.0 rstatix_0.7.0
[49] polspline_1.1.19 MASS_7.3-53.1 zoo_1.8-9 hms_1.0.0 sandwich_3.0-0 RColorBrewer_1.1-2
[55] yaml_2.2.1 curl_4.3.1 KMsurv_0.1-5 rpart_4.1-15 latticeExtra_0.6-29 stringi_1.5.3
[61] highr_0.9 zip_2.1.1 rlang_0.4.11 pkgconfig_2.0.3 matrixStats_0.58.0 evaluate_0.14
[67] labeling_0.4.2 htmlwidgets_1.5.3 tidyselect_1.1.1 R6_2.5.0 generics_0.1.0 multcomp_1.4-17
[73] DBI_1.1.1 pillar_1.6.0 haven_2.4.1 withr_2.4.2 abind_1.4-5 nnet_7.3-14
[79] modelr_0.1.8 crayon_1.4.1 car_3.0-10 survMisc_0.5.5 utf8_1.2.1 rmarkdown_2.7
[85] jpeg_0.1-8.1 data.table_1.14.0 reprex_2.0.0 digest_0.6.27 xtable_1.8-4 munsell_0.5.0
[91] viridisLite_0.4.0
