# Supplementary material for: Phase Ib dose-escalation study of the hypoxia-modifier Myo-inositol trispyrophosphate in patients with hepatopancreatobiliary tumors
Source: Nat Commun. 2021 Jun 21;12:3807. doi: 10.1038/s41467-021-24069-w (PMC8217170; doi:10.1038/s41467-021-24069-w)
Supplement: Supplementary file 5 — Reporting summary [file 41467_2021_24069_MOESM5_ESM.pdf]

nature research

Corresponding author(s): Pierre-Alain Clavien & Pérparim Limani

Last updated by author(s): Apr 24, 2021

Reporting Summary

Nature Research wishes to improve the reproducibility of the work that we publish. This form provides structure for consistency and transparency in reporting. For further information on Nature Research policies, see our Editorial Policies and the Editorial Policy Checklist.

Statistics

For all statistical analyses, confirm that the following items are present in the figure legend, table legend, main text, or Methods section.

n/a	Confirmed	
☐	☒	The exact sample size (n) for each experimental group/condition, given as a discrete number and unit of measurement
☐	☒	A statement on whether measurements were taken from distinct samples or whether the same sample was measured repeatedly
☐	☒	The statistical test(s) used AND whether they are one- or two-sided<br>*Only common tests should be described solely by name; describe more complex techniques in the Methods section.*
☐	☒	A description of all covariates tested
☐	☒	A description of any assumptions or corrections, such as tests of normality and adjustment for multiple comparisons
☐	☒	A full description of the statistical parameters including central tendency (e.g. means) or other basic estimates (e.g. regression coefficient) AND variation (e.g. standard deviation) or associated estimates of uncertainty (e.g. confidence intervals)
☐	☒	For null hypothesis testing, the test statistic (e.g. F, t, r) with confidence intervals, effect sizes, degrees of freedom and P value noted<br>*Give P values as exact values whenever suitable.*
☒	☐	For Bayesian analysis, information on the choice of priors and Markov chain Monte Carlo settings
☒	☐	For hierarchical and complex designs, identification of the appropriate level for tests and full reporting of outcomes
☐	☒	Estimates of effect sizes (e.g. Cohen's d, Pearson's r), indicating how they were calculated

Our web collection on statistics for biologists contains articles on many of the points above.

Software and code

Policy information about availability of computer code

Data collection	secuTrial® (V4.9.1.14, Berlin, Germany; licensed by the clinical trial center of the university of Zurich) was used for protected, monitored and version-controlled data capturing during the clinical trial. Microsoft® Excel® (Microsoft 365 Enterprise, Redmond, Washington/US) was used for data export of secuTrial® and storage for subsequent analysis.
Data analysis	R V 4.0.2 and R-Studio V1.3.1093 were used for all statistical analyses, calculations, and graphical representations.<br>ImageJ (V1.53c, National Institutes of Health, USA) was used for all histological analyses.

For manuscripts utilizing custom algorithms or software that are central to the research but not yet described in published literature, software must be made available to editors and reviewers. We strongly encourage code deposition in a community repository (e.g. GitHub). See the Nature Research guidelines for submitting code & software for further information.

Data

Policy information about availability of data

All manuscripts must include a data availability statement. This statement should provide the following information, where applicable:

- Accession codes, unique identifiers, or web links for publicly available datasets
- A list of figures that have associated raw data
- A description of any restrictions on data availability

All data underlying the calculations and figures in the study are available in the attached source data file. Extended anonymized patient baseline, follow up, and pharmacokinetic information are available in Supplementary Tables 1-3.
Detailed patient-related study raw data (e.g. radiological imaging, laboratory value reports, medical letters, etc. containing patient identifiers such as names, date of birth, addresses or affiliated institutions) which could compromise protection of privacy of research participants are not publicly available due to privacy restrictions. These data are available in anonymized form from the corresponding authors (P.-A. C. or P.L.) upon reasonable request.

Field-specific reporting

Please select the one below that is the best fit for your research. If you are not sure, read the appropriate sections before making your selection.

- [x] Life sciences
- [ ] Behavioural & social sciences
- [ ] Ecological, evolutionary & environmental sciences

For a reference copy of the document with all sections, see nature.com/documents/nr-reporting-summary-flat.pdf

Life sciences study design

All studies must disclose on these points even when the disclosure is negative.

Sample size	Cohort size was based on the traditional 3+3 dose-escalation scheme for phase I studies without formal sample size calculation.
Data exclusions	28 patients included, baseline characterstics and pharmacokinetic of all patients are provided.<br><br>One premature study dropout of 1 patient occurred in cohort 1 due to rapid oncological progression after application of two infusions. As prespecified in the study protocoll, an interval of missed appointments of >7 days between two ITPP applications or an overall of <5 ITPP applications within 5 weeks resulted in the discontinuation of the participant with a replacement by another patient in the same cohort. Data of this patient are therefore excluded for response and efficacy analyses and were not assessed for cohort progression. A replacement for this patient in the same cohort (cohort 1) was recruited (4 patients in cohort 1).
Replication	No biological replicates performed in this human phase Ib dose-escalation study.<br>Serum measurements were performed as technical duplicates with consistent results.<br>Radiological examinations performed as unicates, replication was not ethically justifiable due to radiation exposure.
Randomization	As usual for an exploratory, open-labeled phase Ib dose-escalation study, treatment allocation was not randomized. Covariates were not controlled for.
Blinding	As custom for an exploratory, open-labeled phase Ib dose-escalation study testing safety and tolerability of a new drug , neither investigators nor patients were blinded regarding the study treatment.

Reporting for specific materials, systems and methods

We require information from authors about some types of materials, experimental systems and methods used in many studies. Here, indicate whether each material, system or method listed is relevant to your study. If you are not sure if a list item applies to your research, read the appropriate section before selecting a response.

Materials & experimental systems

n/a	Involved in the study	
[ ]	[x]	Antibodies
[x]	[ ]	Eukaryotic cell lines
[x]	[ ]	Palaeontology and archaeology
[x]	[ ]	Animals and other organisms
[ ]	[x]	Human research participants
[ ]	[x]	Clinical data
[x]	[ ]	Dual use research of concern

Methods

n/a	Involved in the study	
[x]	[ ]	ChIP-seq
[x]	[ ]	Flow cytometry
[x]	[ ]	MRI-based neuroimaging

Antibodies

Antibodies used	Samples were collected in 4% buffered formaldehyde, dehydrated, embedded in paraffin and cut into 5µm sections and stained after antigen retrieval. Hematoxylin/eosin and Masson's trichrome stains were performed according to standard protocols. The following antibodies were used for immunohistochemical staining:<br><br>CkpanB<br>Manufacturer: Dako, M3515<br>Antigen Retrieval: TrisEDTABorat-Buffer, 24min<br>Dilution: 1/50<br>Staining Machine: BenchmarkUltra, Ventana<br>Detection: OptiView DAB Kit<br><br>HIF1a<br>Manufacturer: Abcam, ab16066<br>Antigen Retrieval: TrisEDTABorat-Buffer, 60min

Dilution: 1/400
Staining Machine: Bond III, Leica
Detection: Bond Refine DAB Kit

HIF2a
Manufacturer: Abcam, ab199
Antigen Retrieval: TrisEDTABorat-Buffer, 60min
Dilution: 1/50
Staining Machine: Bond III, Leica
Detection: Bond Refine DAB Kit

CA9
Manufacturer: Abcam, ab15086
Antigen Retrieval: TrisEDTABorat-Buffer, 24min
Dilution: 1/3000
Staining Machine: Bond III, Leica
Detection: Bond Refine DAB Kit

GLUT1 /SLC2A1
Manufacturer: Millipore, 07-1401
Antigen Retrieval: TrisEDTABorat-Buffer, 24min
Dilution: 1/1000
Staining Machine: BenchmarkUltra, Ventana
Detection: OptiView DAB Kit

PECAM1 / CD31
Manufacturer: Dako, M0823
Antigen Retrieval: TrisEDTABorat-Buffer, 24min
Dilution: 1/10
Staining Machine: BenchmarkUltra, Ventana
Detection: OptiView DAB Kit

ERG
Manufacturer: Roche, 790-4576
Antigen Retrieval: TrisEDTABorat-Buffer, 32min
Dilution: prediluted
Staining Machine: BenchmarkUltra, Ventana
Detection: OptiView DAB Kit

VIM
Manufacturer: Dako, M7020
Antigen Retrieval: TrisEDTABorat-Buffer, 16min
Dilution: 1/250
Staining Machine: BenchmarkUltra, Ventana
Detection: OptiView DAB Kit

Validation

CkpanB (Dako, M3515):
AE1/AE3 is a cocktail of two monoclonal antibodies that were obtained by immunizing mice with human callus keratins. AE1/AE3 has been shown to identify the majority of human cytokeratins and thus may be used as a tool for the positive IHC identification of cells of simple and stratified epithelial origin. Antibody AE1 immunoreacts with an antigenic determinant present on most of the subfamily A cytokeratins, including cytokeratins with Moll's designation 10, 13, 14, 15 16 and 19 (MWs of 56.5, 54', 50, 50', 48 and 40 kDa, respectively) but not on Nos. 12, 17 and 18 (55, 47 and 45 kDa). Antibody AE3 reacts with an antigenic determinant shared by the subfamily B cytokeratins including Nos. 1 and 2, 3, 4, 5, 6, 7 and 8 (MWs of 65, 67, 64, 59, 58, 56, 54 and 52 kDa, respectively)
Source: https://www.agilent.com/en/product/immunohistochemistry/antibodies-controls/primary-antibodies/cytokeratin-(concentrate)-76562#productdetails

HHIF1a (Abcam, ab16066):
Immunogen to recombinant fragment corresponding to Human HIF-1 alpha aa 530-826 (C terminal).
Source: https://www.abcam.com/hif-1-alpha-antibody-mgc3-ab16066.html?productWallTab=ShowAll

HIF2a (Abcam, ab199):
Immunogen to synthetic peptide corresponding to Mouse HIF-2-alpha aa 632-646.
Source: https://www.abcam.com/hif-2-alpha-antibody-ab199.html

CA9 (Abcam, ab15086):
Immunogen to synthetic peptide corresponding to Human Carbonic Anhydrase 9/CA9 aa 359-459
Source: https://www.abcam.com/carbonic-anhydrase-9ca9-antibody-ab15086.html

GLUT1 /SLC2A1 (Millipore, 07-1401):
Immunogen to synthetic peptide corresponding amino acids with the C-terminus of human GLUT-1 coupled to KLH (C-ELFHPLGADSQV)
Source: https://www.merckmillipore.com/CH/de/product/Anti-GLUT-1-Antibody-CT,MM_NF-07-1401

PECAM1 / CD31 (Dako, M0823):
Immunogen to cell membrane preparation from the spleen of a patient with hairy cell leukemia.
Source: https://www.agilent.com/en/product/immunohistochemistry/antibodies-controls/primary-antibodies/cd31-endothelial-cell-(concentrate)-76539

ERG (Roche, 790-4576):
Anti-ERG (EPR3864) Rabbit Monoclonal Primary Antibody (anti-ERG (EPR3864)) is directed against the C-terminus of the ETS transcription regulator, ERG, and is capable of detecting both wildtype ERG and truncated ERG resulting from ERG gene rearrangement. This antibody exhibits a nuclear staining pattern and may be used to aid in the identification of prostate adenocarcinomas through the detection of truncated ERG. The antibody is intended for qualitative staining in sections of formalin-fixed, paraffin-embedded tissue
Source: https://diagnostics.roche.com/content/dam/diagnostics/us/en/resource-center/Tissue-product-catalog-2019.pdf

VIM (Dako, M7020):
Immunogen to vimentin isolated from bovine eye lens.
Source: https://www.agilent.com/cs/library/packageinsert/public/SSM7020CEEFG_01.pdf

Human research participants

Policy information about studies involving human research participants

Population characteristics

28 patients (18 males and 10 females) with a median age of 65 years (IQR: 53-69) were included in the study between 04/27/2015 to 07/06/2018. Patients suffered from PDAC (n=10), colorectal cancer liver metastases (CRLM, n=8), CCA (n=7), and HCC (n=3). 25/28 of patients had received extensive previous anti-tumor therapies (median of two regimens, IQR 1-4) prior to study inclusion, with a median of two involved organs at study start (IQR 1-4).

Recruitment

Patients were being recruited and enrolled at the University Hospital Zurich (USZ).

The enrollment/recruitment process preceeded as follows:

Patients referred to/treated at the surgical or oncological outpatient department of the university hospital of Zurich and fulfilling the information criteria (aged ≥18 years, diagnosed with irresectable HPB tumors, including CRLM, PDAC, HCC and CCA, Eastern Cooperative Oncology Group performance status score ≤1, adequate hematological, renal, and hepatic function, at least 28 days recovery from recent surgery or chemo- or radiotherapy), were informed about the trial by their treating physician if no curative option was available.

If patients were interested, an informative talk was following conducted with a physician investigator, outlining the study setup, aims of the trial as well as potential benefits and complications of study participation. Care was taken make patients aware of the limitations and pitfalls (e.g. lack of efficacy, no curative potential, potential side effects) of a phase Ib trial.

If patients temporary agreed to take part in the trial (normally discussed over phone at least 24 hours after the first talk), the case of the patient was presented and discussed at the multidisciplinary HPB tumor board at the university hospital of Zurich, including at least specialized 2 consultants of each: HPB oncological surgeons, GI oncologists, gastroenterologists, radio-oncologists and abdominal pathologists.

Patients were cleared for study inclusion, if all disciplines/participants agreed. Major objections of one discipline/participant lead to patients not being directly recruited, but being referred to discuss standard therapy or other treatment options first. If cleared, patients were re-seen at the outpatient clinical (normally 1 week after primary visit) for another information round with answering of remaining questions and subsequent signature of the informed consent.

Study preparations were then set in place (e.g. prepartion of ITPP), necessary pre-ITPP imaging organized and treatment started within 5-10 days (treatment normally was administered mondays - wednesdays - fridays for 3 weeks).

Potential biases during patient recruitment: None

Ethics oversight

The study protocol was approved by the ethics committee of Zurich (KEK-ZH-Nr. 2014-0374) and the national regulatory authority Swissmedic (2015DR1009). The study, including patient recruitment and data collection, was continuously monitored by uninvolved clinical trial managers of the clinical trials center of the university of Zurich. The study was audited twice by external reviewers during the phase of patient recruitment, which objected no relevant irregularities.

Note that full information on the approval of the study protocol must also be provided in the manuscript.

Clinical data

Policy information about clinical studies

All manuscripts should comply with the ICMJEguidelines for publication of clinical research and a completedCONSORT checklist must be included with all submissions.

Clinical trial registration	ClinicalTrials.gov NCT02528526
Study protocol	The essential parts of the study protocol have been translated into english and are available in the supplementary information.
Data collection	Patients were treated in the study between 04/27/2015 to 07/06/2018. Data was collected by specifically trained physician investigators and study nurses at the phase 1 unit of the clinical trials center of the University of Zurich and entered into SecuTrial (see above).
Outcomes	Primary objetive was assessment of safety and patient tolerance as measured by collection of adverse effect information.<br>Primary outcome were (i) assessment of the safety and tolerability of increasing doses of ITPP, and (ii) establishment of the MTD (primary endpoint) according to the dose escalation schema as measured by collection of adverse effects information according to Common Terminology Criteria for Adverse Events (CTCAE, US National Cancer Institute, version 4.03).<br><br>Secondary objectives included assessment of (a) pharmacokinetics, (b) antitumor activity via radiological assessment, and (c) assessment of circulatory tumor-specific and angiogenic markers.<br>Secondary Outcomes: The pharmacokinetics of increasing doses of ITPP administration was measured using repeated blood measurements (plasma). Pharmacokinetic parameters assessed include (i) area under the curve (AUC) of the concentration versus time curve from time zero to the last measurable concentration, (ii) AUC from time zero extrapolated to infinity maximum observed concentration (Cmax), (iii) time of observed Cmax, (iv) trough serum concentration, (v) total body clearance, (vi) terminal elimination half-life (t1/2), and (vii) volume of distribution at steady state.<br>The efficacy of ITPP monotherapy and subsequent chemotherapy was measured by radiological assessment, with imaging to be performed before and after ITPP therapy, as well as after chemotherapy, if possible, using fluorodeoxyglucose F18 PET (response evaluated by European Organisation for Research and Treatment of Cancer criteria) and MRI (response evaluated by RECIST 1.1); and by biochemical assessment, using measurement of specific tumor and angiogenesis markers in serum.
